# Performance Limits and Advancements in Single 2D Transition Metal Dichalcogenide Transistor

**DOI:** 10.1007/s40820-024-01461-x

**Published:** 2024-08-09

**Authors:** Jing Chen, Ming-Yuan Sun, Zhen-Hua Wang, Zheng Zhang, Kai Zhang, Shuai Wang, Yu Zhang, Xiaoming Wu, Tian-Ling Ren, Hong Liu, Lin Han

**Affiliations:** 1 Institute of Marine Science and Technology, Shandong University, Qingdao, 266237 Shandong People’s Republic of China; 2 School of Integrated Circuits and Beijing National Research Center for Information Science and Technology (BNRist), Tsinghua University, Beijing, 100084 People’s Republic of China; 3 BNRist, Tsinghua University, Beijing, 100084 People’s Republic of China; 4 State Key Laboratory of Crystal Materials, Shandong University, Jinan, 250100 Shandong People’s Republic of China; 5 Shenzhen Research Institute of Shandong University, Shenzhen, 518057 People’s Republic of China; 6 Shandong Engineering Research Center of Biomarker and Artificial Intelligence Application, Jinan, 250100 People’s Republic of China

**Keywords:** Two-dimensional transistors, Dimension limits, Performance limits, Memory devices, Artificial synapses

## Abstract

Two-dimensional (2D) transition metal dichalcogenides (TMDs) allow for atomic-scale manipulation, challenging the conventional limitations of semiconductor materials. This capability may overcome the short-channel effect, sparking significant advancements in electronic devices that utilize 2D TMDs. Exploring the dimension and performance limits of transistors based on 2D TMDs has gained substantial importance. This review provides a comprehensive investigation into these limits of the single 2D-TMD transistor. It delves into the impacts of miniaturization, including the reduction of channel length, gate length, source/drain contact length, and dielectric thickness on transistor operation and performance. In addition, this review provides a detailed analysis of performance parameters such as source/drain contact resistance, subthreshold swing, hysteresis loop, carrier mobility, on/off ratio, and the development of p-type and single logic transistors. This review details the two logical expressions of the single 2D-TMD logic transistor, including current and voltage. It also emphasizes the role of 2D TMD-based transistors as memory devices, focusing on enhancing memory operation speed, endurance, data retention, and extinction ratio, as well as reducing energy consumption in memory devices functioning as artificial synapses. This review demonstrates the two calculating methods for dynamic energy consumption of 2D synaptic devices. This review not only summarizes the current state of the art in this field but also highlights potential future research directions and applications. It underscores the anticipated challenges, opportunities, and potential solutions in navigating the dimension and performance boundaries of 2D transistors.

## Highlights

- The review provides a comprehensive summary of performance limits of the single two-dimensional transition metal dichalcogenide (2D-TMD) transistor.
- The review details the two logical expressions of the single 2D-TMD logic transistor, including current and voltage.
- The review demonstrates the two calculating methods for dynamic energy consumption of 2D synaptic devices.

## Graphical abstract

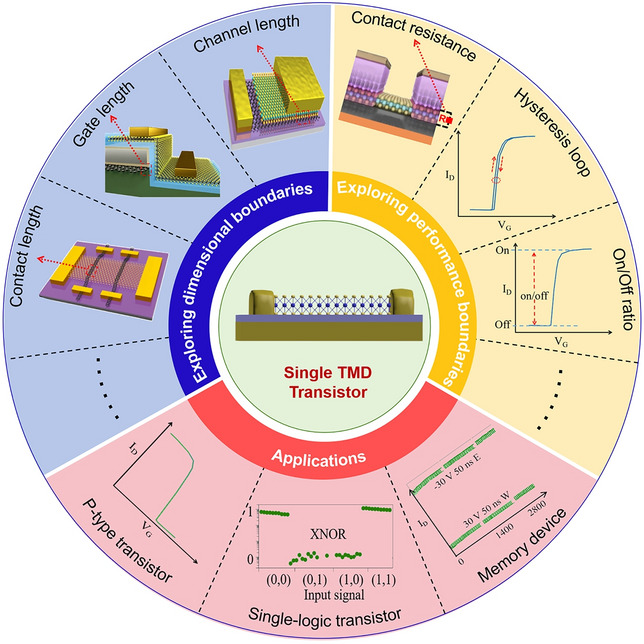

## Introduction

Transistor technology has been instrumental in driving the progress of contemporary electronics. From the invention of the first point-contact transistor in 1947 to the evolution of silicon-based MOSFETs, this technology has consistently evolved to meet the escalating demand for more compact, speedier, and energy-efficient devices. In the past decade, a novel class of materials, known as two-dimensional (2D) transition metal dichalcogenides (TMDs), has emerged as a promising candidate for future-generation transistors [1–5]. The evolution and significance of transistors based on 2D TMDs have become intriguing topics in recent years, owing to their unique properties and potential applications across various disciplines [6]. TMDs, encompassing molybdenum disulfide (MoS_2_), molybdenum telluride (MoTe_2_), and tungsten diselenide (WSe_2_), possess a layered structure, paving the way for atomic scale transistor development, a considerable advancement from traditional semiconductor materials [7, 8]. Furthermore, the non-zero bandgap of TMDs make them particularly suited for applications in the semiconductor industry, where device miniaturization and performance enhancement are paramount. 2D monolayer TMDs also possess a direct bandgap, which is beneficial for optoelectronic applications [9]. Their mechanical flexibility and chemical stability open avenues for flexible and wearable electronics [10–13]. The thin nature of 2D TMDs permits the creation of van der Waals heterostructures by stacking varying 2D materials, thus enabling the design of devices with tailor-made properties [14–16]. Nevertheless, realizing the potential of 2D TMDs in transistor technology still exists challenges. Issues such as contact resistance, variability, and material quality must be addressed to fully capitalize on their benefits [17, 18].

TMDs, with their unique properties and the possibility of manipulating dimensions at the atomic scale, unlock novel opportunities for fabricating ultrascaled, high-performance devices. While the journey of 2D TMDs-based transistors is just beginning, their potential to drive the next revolution in electronics is undeniable. The exploration of these materials holds significance not only for the evolution of transistor technology, but also for the wider field of nanotechnology.

### The Limitations of Traditional Semiconductor Materials and the Potential of 2D TMDs to Address These

**Fig. 1:**
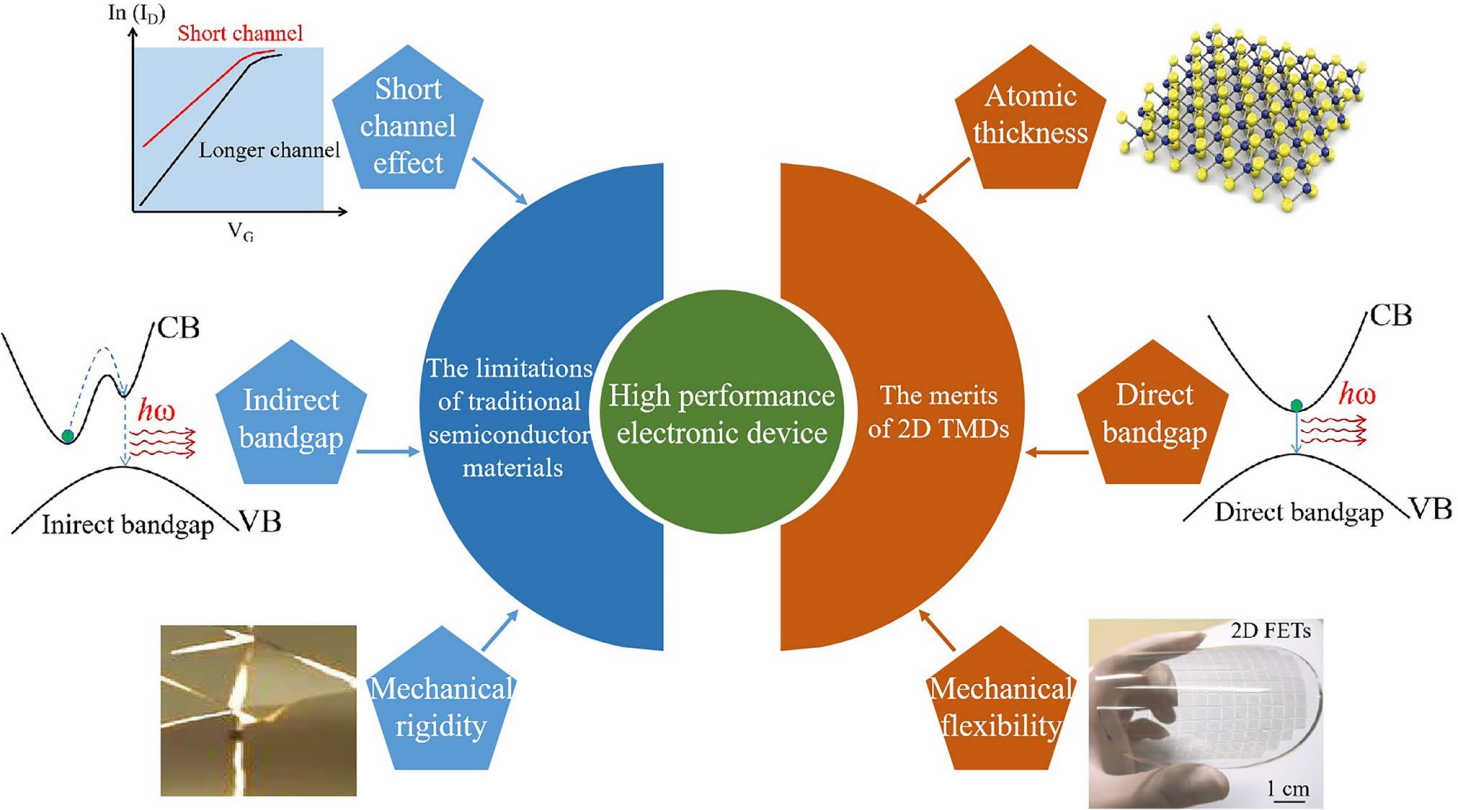
The limitations of traditional semiconductor materials and the merits of 2D TMDs for high performance electronic device. The diagram of flexible 2D FETs. Reproduced with permission. Reference [10] Copyright 2022, Springer Nature

Traditional semiconductor materials, most notably silicon (Si), have been the bedrock of contemporary electronics. However, as the industry strives for device performance enhancement and size reduction, several inherent limitations of these materials have surfaced [19] (Fig. 1). A significant challenge is the short-channel effect, which arises when the channel length of the transistor is diminished. As this length decreases, the control over current flow is compromised, leading to increased leakage current, power dissipation, and diminished reliability. Another constraint is Si's indirect bandgap, which hinders its application in optoelectronic devices. The indirect bandgap makes efficient light emission challenging, hindering the development of Si-based light-emitting diodes or lasers. Furthermore, Si's mechanical rigidity limits its applicability in flexible electronics. The burgeoning demand for wearable and flexible devices necessitates the introduction of new materials that can endure mechanical strain [20].

2D TMDs offer promising solutions to these constraints (Fig. 1). The emergence of 2D materials has spurred a revolution in nanoelectronics over the past decade. These materials hold tremendous potential for the advancement of next-generation semiconductor devices [21]. Due to their atomic thickness, 2D TMDs provide superior control over the channel, potentially mitigating the short-channel effects and facilitating further device miniaturization [1, 8]. Unlike Si, many 2D monolayer TMDs, such as MoS_2_ and WSe_2_, possess a direct bandgap, making them suitable for optoelectronic applications [9]. Moreover, 2D TMDs exhibit exceptional mechanical flexibility, rendering them promising candidates for the upcoming generation of flexible and wearable electronics [10–12, 22]. The capability of 2D TMDs to endure mechanical deformation without losing their electronic properties gives them a significant advantage over traditional semiconductors. Additionally, machine learning-based predictive models have been employed to predict 2D materials' properties, including magnetic ordering, bandgap properties, and point defect designs. This allows for the screening and determination of optimal synthesis mechanisms for such materials [23].

As a result, 2D TMDs offer a promising path to circumvent the limitations of traditional semiconductor materials. Their unique properties and the possibility to manipulate dimensions at an atomic scale offer new opportunities for the development of the next generation of ultra-scaled high-performance devices. Despite these promising characteristics, the implementation of 2D TMDs in transistors comes with its own set of challenges, including material synthesis, device fabrication, and the control of electrical properties [18, 24]. However, ongoing research and technological advancements are progressively pushing the boundaries, paving the way for the realization of 2D TMD-based transistors.

### The Relevance of Dimension and Performance Constraints in the Context of Technological Progress

The significance of dimension and performance constraints in the context of technological progress is a crucial area of research in the electronics and optoelectronics field. The relentless push for technological advancement in the electronics industry has been guided by Moore's Law [25]. This principle has driven the miniaturization of transistors, a vital factor in developing faster, more powerful, and more energy-efficient devices. As the size of transistors nears the atomic scale, dimension and performance constraints have increasingly emerged as significant challenges [26]. The miniaturization process is beginning to confront physical limitations such as quantum effects, increased leakage current, power dissipation, and reduced reliability due to short-channel effects.

**Fig. 2:**
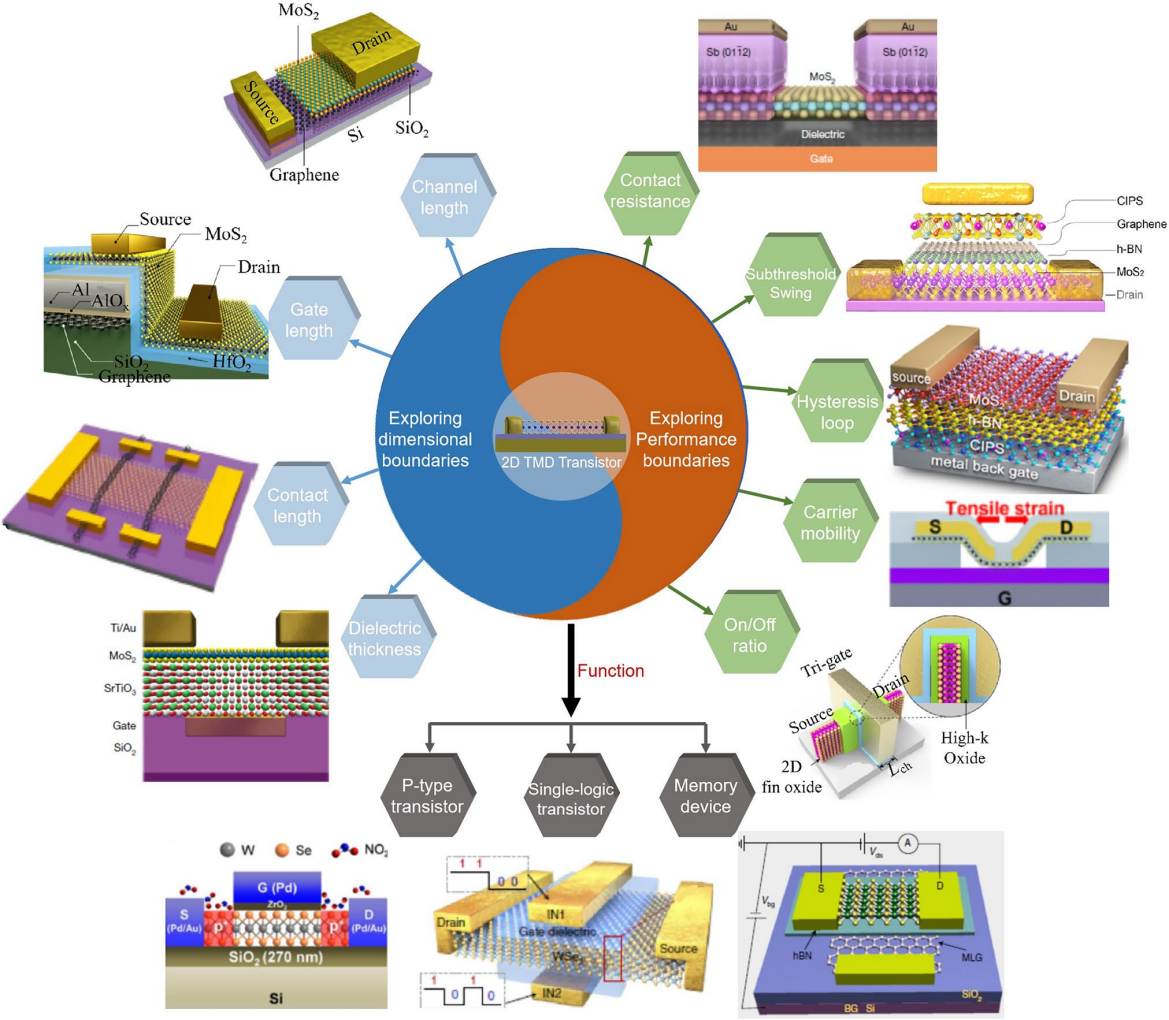
Demonstration of dimension and performance limits in 2D TMD transistors. Channel length diagram. Reproduced with permission. Reference [27] Copyright 2021, Springer Nature. Gate length diagram. Reproduced with permission. Reference [28] Copyright 2022, Springer Nature. Contact length diagram. Reproduced with permission. Reference [29] Copyright 2023, Springer Nature. Dielectric thickness diagram. Reproduced with permission. Reference [30] Copyright 2022, Springer Nature. Contact resistance diagram. Reproduced with permission. Reference [31] Copyright 2023, Springer Nature. Subthreshold swing diagram. Reproduced with permission. Reference [32] Copyright 2021, Springer Nature. Hysteresis loop diagram. Reproduced with permission. Reference [33] Copyright 2023, American Chemical Society. Carrier mobility diagram. Reproduced with permission. Reference [34] Copyright 2023, American Chemical Society. On/Off ratio diagram. Reproduced with permission. Reference [35] Copyright 2023, Springer Nature. P-type transistor diagram. Reproduced with permission. Reference [36] Copyright 2022, Wiley–VCH Verlag. Single-logic transistor diagram. Reproduced with permission. Reference [37] Copyright 2021, Springer Nature. Memory device diagram. Reproduced with permission. Reference [38] Copyright 2021, Springer Nature

The dimension constraint refers to the physical limits of device miniaturization, such as channel length, gate length, source/drain contact length, and dielectric thickness (Fig. 2), which influence their performance. The continuous reduction of transistor dimensions has reached a point where further shrinkage could disrupt the device's structural integrity and operational functionality. For example, reducing the channel length can enhance the speed of the transistor, but may also increase leakage current, leading to higher power consumption. Similarly, reducing the gate length can improve the transistor’s switching speed but may increase the risk of short-channel effects, which can degrade the device’s performance. The performance constraint involves maintaining or enhancing the device's operational efficiency, speed, and power consumption as its size is reduced. This is a major challenge as traditional semiconductor materials like silicon struggle to maintain their performance when scaled down to a few nanometers [19]. Performance constraints such as source/drain contact resistance, subthreshold swing, hysteresis loop, carrier mobility, and on/off ratio also significantly impact the overall performance of the transistor (Fig. 2). For example, reducing the source/drain contact resistance can enhance the transistor's current drive capability, while reducing the subthreshold swing can improve the transistor’s energy efficiency.

In terms of technological progress, overcoming these dimension and performance constraints is essential for developing next-generation electronic devices. For example, 2D TMD-based transistors demonstrate great potential in overcoming these constraints due to their unique properties such as tunable bandgap and high carrier mobility [1]. Their atomic-scale thickness allows for excellent electrostatic control, which could alleviate short-channel effects and enable further device miniaturization [8]. Furthermore, the direct bandgap and high on/off current ratio properties of 2D monolayer TMDs offer promising performance characteristics that could enhance device function [9]. However, there are still many challenges to be addressed, such as high contact resistance and the presence of traps in the 2D TMDs, which can degrade the device’s performance. Therefore, further research is needed to fully understand and overcome these challenges [18]. In conclusion, addressing these dimension and performance constraints is a critical area of study that can pave the way for next-generation electronic devices development and is essential to sustain the technological progress that society has become accustomed to. The exploration of novel materials like 2D TMDs is an integral part of this journey, marking a significant shift in the landscape of semiconductor technology [24].

### Outline of the Review’s Scope, Objectives, and Structure

This review aims to offer a comprehensive overview of the present state of research and development within the field of 2D TMD-based transistors. The aim is to delve into the implications of 2D TMDs on transistor dimensionality and performance, providing a profound understanding of these topics and contributing to the ongoing discourse around the future of semiconductor technology. The scope of the review encompasses a thorough examination of the dimensional limits of transistors based on 2D TMDs, including channel length miniaturization, gate length reduction, source/drain contact length minimization, and dielectric thickness reduction. This review will also explore the performance limits of these transistors, focusing on aspects such as the reduction of source/drain contact resistance, subthreshold swing reduction, hysteresis loop reduction, carrier mobility enhancement, on/off ratio enhancement, and the realization of p-type and single logic transistors, and memory devices. As part of the exploration of the dimension and performance limits of transistors utilizing 2D TMDs, strategies in traditional semiconductor transistors and those in 2D transistors will be compared to highlight the advantages of 2D TMDs.

The structure of the review will be methodological and systematic, divided into six main sections. The introduction provides a brief overview of the evolution and significance of transistors based on 2D TMDs and outlines the scope, objectives, and structure of the review. Subsequent sections delve into understanding 2D TMDs, exploring the dimensional and performance limits of transistors based on 2D TMDs, the role and significance of p-type and single-logic transistors, and the specific challenges and opportunities associated with using 2D TMDs for memory devices. The review concludes with a summary of the key points discussed and an outlook on future prospects and potential directions in the field of 2D transistors. The objective of this review is to help readers gain a comprehensive understanding of the current state of research in the field of 2D TMD-based transistors, the challenges and opportunities associated with their dimensional and performance limits, and the potential impact of overcoming these limits on future technological advancements.

## Two-Dimensional TMDs and Their Implications for Dimensionality of Electronic Devices

2D TMDs have caught the attention of researchers due to their unique layered structure, which carries significant implications for dimensionality. The layered structure of 2D TMDs allows for potential manipulation of dimensions at the atomic scale, facilitating the fabrication of devices with dimensions unreachable with traditional semiconductors [39].

### Unique Layered Structure of 2D TMDs and Its Implications for Dimensionality

**Fig. 3:**
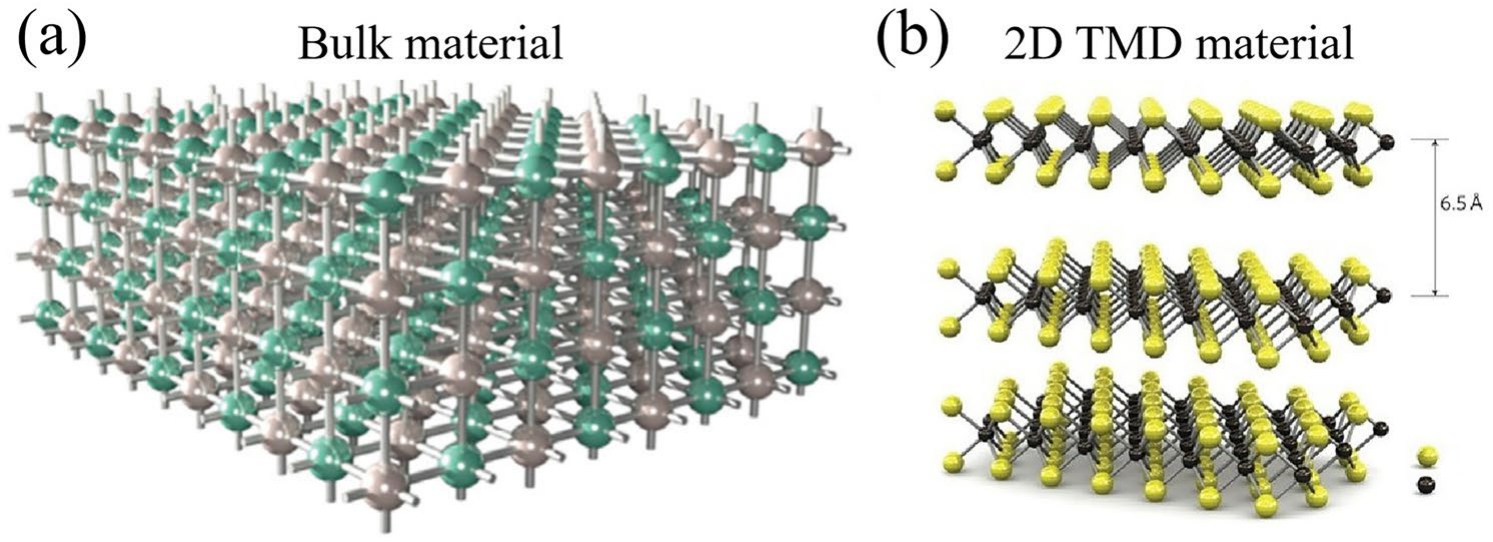
Comparison of conventional bulk material and 2D TMD material. **a** Bulk material. Reproduced with permission. Reference [1] Copyright 2022, Elsevier. **b** 2D TMD material. Reproduced with permission. Reference [39] Copyright 2011, Springer Nature

As shown in Fig. 3, unlike traditional bulk semiconductors (Fig. 3a), 2D TMDs consist of a single layer of transition metal atoms (Fig. 3b), such as molybdenum or tungsten, sandwiched between two layers of chalcogen atoms, typically sulfur, selenium, or tellurium [40]. The layered structure of 2D TMDs is characterized by weak interlayer bonding and strong intralayer bonding, which allows for exfoliating single atomic layers [39] or stacking them together in different configurations [41]. This property is pivotal as it enables the manipulation of these materials at the nanoscale, catering to various applications. The interlayer spacing of TMDs is approximately 6.5 Å, and their stacking order is indicated by the stacking index *c* [41]. This tunable interlayer coupling is unique to 2D materials and provides immense opportunities to engineer the electronic properties of devices [14]. The thickness of TMDs determines their band structure, which further underscores the layered structure's importance. Unlike graphene with a zero-gap band structure, 2D TMDs present a tunable bandgap, making them more suitable for specific applications.

The unique electrical properties of 2D TMDs result from their reduced dimensionality, which significantly differs from their 3D counterparts. For instance, TMDs can transform from an indirect bandgap to a direct bandgap when reduced down to monolayers [39]. Their direct bandgap, absent in bulk TMDs and silicon, enables efficient light emission and absorption, making them ideal for applications in optoelectronics [9]. Moreover, the thinness of 2D TMDs allows for excellent electrostatic control, crucial for mitigating short-channel effects in field-effect transistors [8]. The 2D nature of TMDs has implications for mechanical flexibility. The van der Waals forces between layers enable them to slide over each other without breaking, making 2D TMDs remarkably flexible and robust. These characteristic paves the way for flexible and wearable electronics [10, 11]. However, the 2D nature of TMDs also presents challenges, such as increased sensitivity to environmental conditions and difficulties in fabrication and integration into devices [18].

As a result, the unique layered structure of 2D TMDs showcases the profound impact of dimensionality on material properties, offering a rich platform for exploring new physics and developing next-generation electronic and optoelectronic devices [42]. Nonetheless, further research is needed to fully exploit these materials' potential and overcome the challenges associated with their manipulation at the atomic scale.

### Layered Structure Allowing for Potential Manipulation of Dimensions at Atomic Scale

Control over thickness has significant implications for the electronic properties of 2D TMDs, as it allows for tuning the bandgap and other vital electronic parameters [9]. The weak van der Waals forces in 2D TMDs also enable flexibility in stacking these materials. Different layers can be arranged in various sequences and orientations to form van der Waals heterostructures [41]. These heterostructures provide a new degree of freedom in designing electronic and optoelectronic devices, as they allow for the combination of materials with different electronic properties in a single device [15]. Additionally, the weak interlayer forces enable mechanical exfoliation or layer transfer techniques, allowing the construction of complex heterostructures and superlattices with atomic precision [43]. This capacity to engineer and manipulate the dimensions of 2D TMDs at the atomic scale introduces new possibilities for fabricating desired nanoscale devices [1].

Furthermore, atomic-scale structural modification of 2D TMDs can be achieved through various strategies like direct introduction during synthesis, post-treatment, chemical potential control, lattice plane control, molecular assembly, and anisotropic etching [44]. These modifications can lead to changes in the atomic-scale structures of materials, including edge structures, atomic defects [45], grain boundaries [46]. These modifications can result in changes in the electrical properties of 2D materials, essential for nanoscale devices [44].

However, manipulating dimensions at the atomic scale still presents significant challenges, such as the need for precise control over material synthesis and device fabrication processes [18]. Furthermore, the as-obtained 2D materials during fabrication and post-treatment may lead to possible unexpected phase transitions or reductions in their crystallinity, degrading the performance of 2D devices [44].

**Fig. 4:**
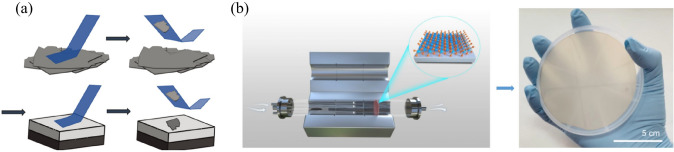
Fabrication methods of monolayer TMDs. **a** Micromechanical exfoliation method. Reproduced with permission. Reference [51] Copyright 2022, Royal Society of Chemistry. **b** Chemical vapor deposition method. Reproduced with permission. Reference [47] Copyright 2022, Wiley

Currently, there are two mainstream methods for fabricating monolayer TMDs such as micromechanical exfoliation (Fig. 4a) and chemical vapor deposition methods [47–50] (Fig. 4b). The layered structure of 2D TMDs enables potential manipulation of dimensions at the atomic scale, opening up exciting possibilities for engineering atomic quantum defects and developing next-generation electronic and optoelectronic devices. Nonetheless, further research is needed to overcome the challenges associated with atomic-scale structural modification and to fully exploit these materials' potential.

## Dimensional Limits of Transistors

**Fig. 5:**
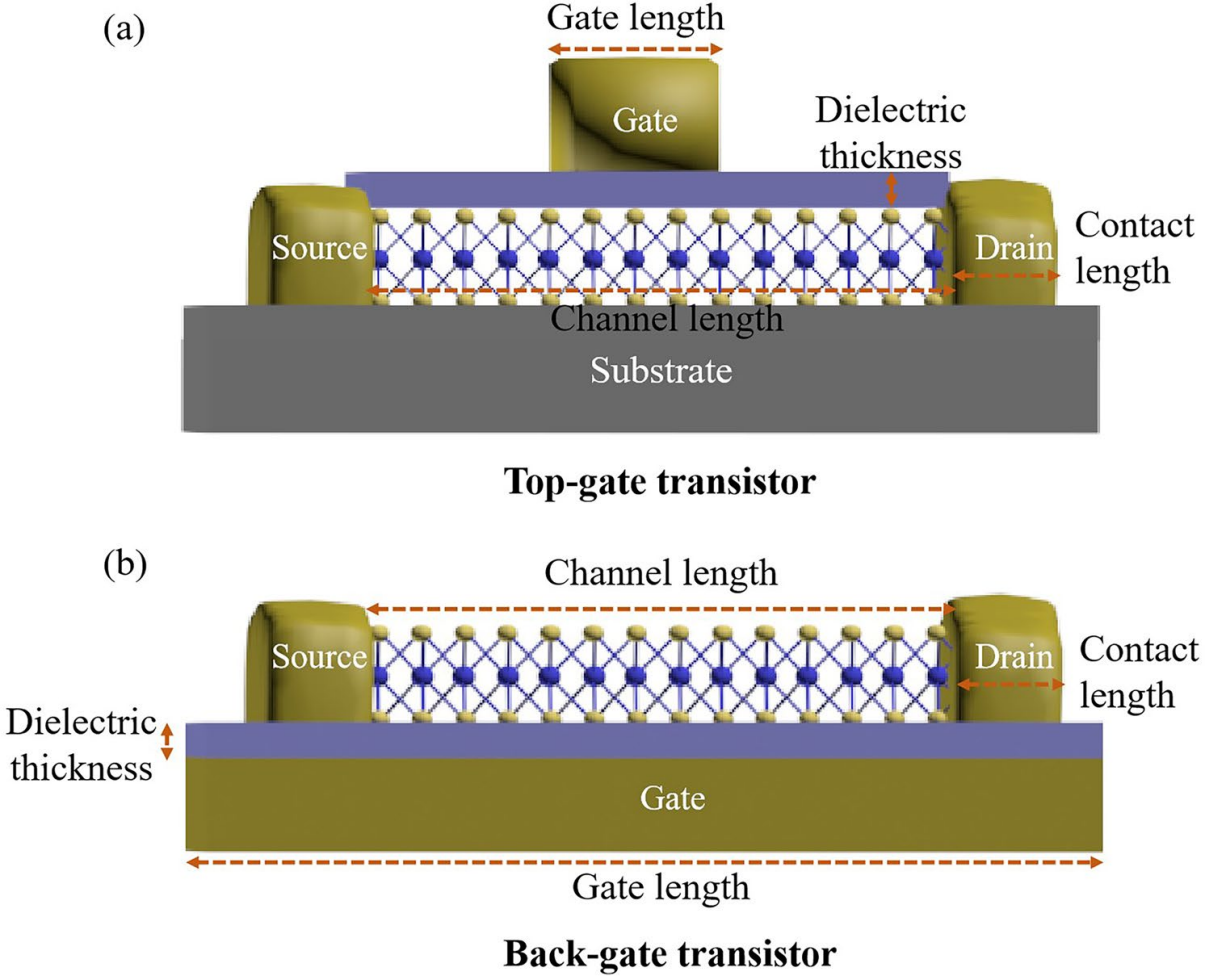
Demonstration of the dimensions of 2D transistors, such as dielectric thickness, channel length, gate length, and contact length. **a** Top-gate transistor. **b** Back-gate transistor

The dimensional parameters of transistors including channel length, gate length, source/drain contact length, and dielectric thickness, which play important role in the electrical characteristics of transistors. The definition of these dimensional parameters in transistors with different structures is presented in Fig. 5.

### Significance of Channel in Transistor Miniaturization

The channel length, defined as the distance between the source and drain of a transistor, significantly impacts the device's performance characteristics. Miniaturizing the channel length is a critical aspect of transistor scaling, which fuels the continuous advancement of semiconductor technology [25]. Reducing the channel length enhances the transistor's switching speed by shortening the distance that carriers must travel. This leads to faster operation and improved device performance [52, 53]. Moreover, miniaturization allows for a higher density of transistors on a chip area, leading to increased computational power and functionality [25]. As channel lengths approach the nanometer scale, several challenges arise due to traditional Si CMOSFETs reaching the miniaturization limit of sub-5 nm technology nodes according to Moore’s Law [1]. Threshold voltage roll-off, drain-induced barrier lowering, and short-channel effects can degrade device performance and increase power consumption [54]. These effects result from the diminishing control of the gate over the channel as the gate length decreases, leading to leakage currents and higher power dissipation [55, 56].

2D TMDs present promising solutions to these challenges. Their atomically thin nature allows for effective gate control even at extremely short channel lengths, mitigating short-channel effects and enabling further miniaturization [1, 39]. The direct bandgap of monolayer TMDs could potentially enable ultra-low power digital and analog circuits [10]. The atomic thickness of 2D TMDs also leads to a reduced scattering rate for carriers, potentially enhancing device performance [57]. The band structure of TMDs, which can transition from an indirect to a direct bandgap in monolayer form, could be beneficial for electronic and optoelectronic applications [58].

Channel length's role in transistor miniaturization is not just about reducing physical size. By manipulating channel length and using novel materials like 2D TMDs, researchers can control transistors' electronic properties, potentially resulting in devices with improved performance, lower power consumption, and greater functionality [36]. The miniaturization of the channel length is a key factor driving transistor scaling and enhancing device performance, 2D TMDs offer promising solutions for further scaling down the channel length for the next generation of nanoscale electronic devices [8, 24]. However, challenges persist in the miniaturization of 2D TMD transistors, including issues related to material synthesis, device fabrication, contact resistance, and environmental stability [59, 60]. Continued research and innovation are required to overcome these challenges and further realize transistor miniaturization using 2D TMDs.

As part of the exploration of the dimension and performance limits of transistors utilizing 2D TMDs, strategies for scaling down the channel length in traditional semiconductor transistors and those in 2D transistors will be compared to highlight the advantages of 2D TMDs.

#### Channel Length Scaling-Down in Traditional Semiconductor Transistors

In the quest to enhance performance and efficiency, the semiconductor industry has pursued relentless transistor channel length scaling. Recent advancements and methodologies have facilitated this miniaturization, pushing technological boundaries and fueling innovation. A notable advancement is the development of the Fin Field-Effect Transistor (FinFET) design. This design has been instrumental in facilitating transistor scaling into the sub-10 nm regime. Unlike planar transistors, FinFETs boast a three-dimensional structure with a thin "fin" of silicon extending into the device, which provides superior gate control and helps reduce short-channel effects [61]. Further, the employment of advanced lithography techniques, like extreme ultraviolet (EUV) lithography, has allowed for precise patterning of smaller features, thus enabling further miniaturization of the channel length [62].

#### Channel Length Scaling-Down in Two-dimensional Transistors

**Fig. 6:**
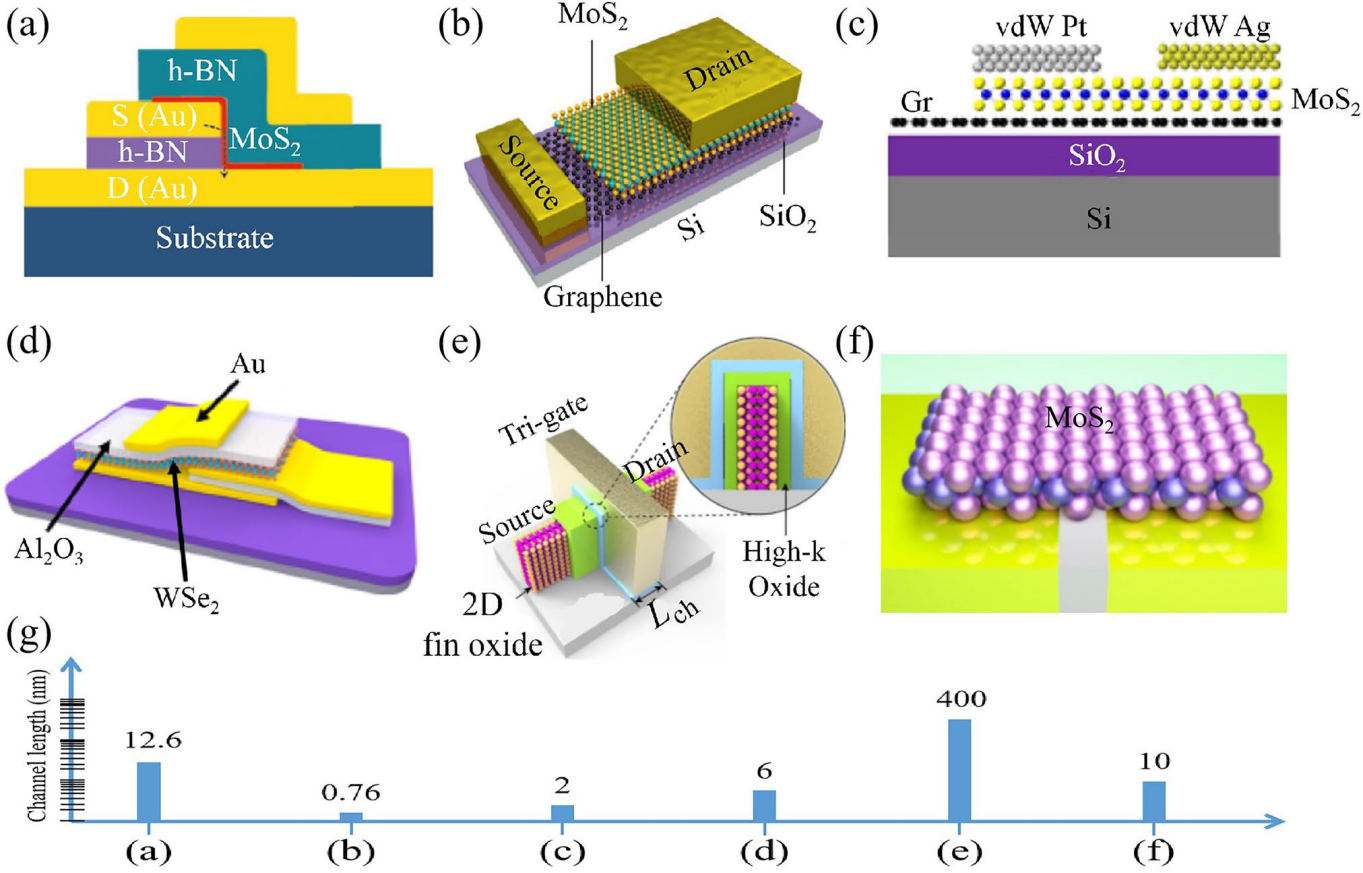
Scaling down the channel length of transistors. **a** 2D vertical-channel transistors. Reproduced with permission. Reference [65] Copyright 2020, Wiley. **b** Vertical transistor with a sub-1-nm channel. Reproduced with permission. Reference [27] Copyright 2021, Springer Nature. **c** Sub-2 nm vertical-channel transistors. Reproduced with permission. Reference [66] Copyright 2023, American Chemical Society. **d** Monolayer WSe_2_ sloping-channel transistors. Reproduced with permission. Reference [67] Copyright 2023, American Chemical Society. **e** 2D fin field-effect transistors. Reproduced with permission. Reference [35] Copyright 2023, Springer Nature. **f** 2D devices with ultraflat sub-10 nm gap electrodes. Reproduced with permission. Reference [68] Copyright 2021, American Chemical Society. **g** Channel lengths of above 2D transistors

Recent advancements in 2D TMDs have opened new avenues for transistor miniaturization (Fig. 6). The atomically thin nature of these materials could potentially mitigate short-channel effects, offering superior gate control even at nanometer channel lengths [1, 21, 63]. Notably, the emergence of monolayer TMD materials like MoS_2_, WS_2_, and WSe_2_ has shown promise in achieving transistors with channel lengths nearing the atomic scale [8], which has been confirmed by theory simulation [64]. Various fabrication methodologies are being studied to effectively scale down 2D TMD transistors.

A significant breakthrough in this field is the creation of vertical transistors based on 2D materials [27, 65, 66, 69–71] (Fig. 6a–c). The first study, conducted by Liting Liu et al. [72], confirmed a MoS_2_ channel between source and drain electrodes in a vertical direction, termed as vertical field-effect transistors. This vertical-transistor configuration exhibits a minimized Fermi-level pinning effect and direct tunneling current. The group successfully fabricated sub-3-nm p-type and n-type vertical transistors using WSe_2_ and WS_2_. Moreover, multi-vertical-transistors can be vertically stacked, laying the foundation for high-density integrated circuits [73]. These vertical transistors’ on/off ratios are limited by a strong source-drain tunneling current in the off state. The van der Waals metal-contact method can be employed for suppressing the off-state tunneling current [66]. Additionally, 2D materials can also be transferred onto the source/drain electrode for sloping-short-channel transistors [67] (Fig. 6d).

The continuous advancement of silicon-based process nodes has the guiding significance for the size reduction and performance improvement of two-dimensional material-based transistors. The concept of FinFET in traditional silicon-based transistors has now been successfully implemented in two-dimensional material transistors. Another major breakthrough is the construction of 2D fin field-effect transistors, integrating single-crystal high-k gate oxide Bi_2_SeO_5_ and semiconductor Bi_2_O_2_Se epitaxially. These transistors demonstrate high electron mobility (μ) of 270 cm^2^ V^−1^ s^−1^, ultralow *I*_OFF_ of 1 pA μm^−1^, high *I*_ON_ of 830 μA μm^−1^, high on/off current ratios (*I*_ON_/*I*_OFF_) of 10^8^ at a 400-nm channel length, providing at a new avenue for extending Moore’s law [35] (Fig. 6e). Figure 6g shows the quantitative comparation of channel lengths. Moreover, the fin-width of Bi_2_O_2_Se fin field-effect transistor can be shrunk down to 1.2 nm [35, 74]. Mao-Lin Chen et al. have constructed monolayer MoS_2_ fin field-effect transistor with sub-1 nm fin-width limit [75]. These results indicate that the channel width of 2D transistors can be shrunk down to approximately 1 nm. Currently, only Bi_2_O_2_Se and MoS_2_ have been employed for constructing FinFET structures in terms of fabrication. In Bi_2_O_2_Se FinFET, the vertical 2D Bi_2_O_2_Se channel was synthesized in homemade CVD systems [35]. While, in MoS_2_ FinFET, the vertical MoS_2_ channel was fabricated through many steps: Si on insulator substrate, 300 nm step, HfO_2_ coating, side wall etching, TMD growth, S–D patterning, Plane removing, Si wet etching, HfO_2_ supported ML-Fin, Top gate patterning [75]. Hence, we think Bi_2_O_2_Se-FinFET process is a viable FinFET process strategy due to its simpler process fabrication.

Statistical results have shown that 2D double-gate Transistors with 30-nm channel length exhibit high performance, further confirming the potential of 2D TMDs in transistor channel length scaling [76]. MoS_2_ transistors with a 3 nm channel length have been realized by the electromigration of metal interconnection, displaying on/off ratios up to 2 × 10^5^ and field-effect mobility up to 33.5 cm^2^ V^−1^ s^−1^ [77]. Ultraflat gap electrodes have been employed to downscale 2D channels to sub-10 nm [68, 77, 78] (Fig. 6f). Despite these advances, scaling down transistors based on 2D TMDs poses challenges. As the channel length reduces, quantum mechanical effects like tunneling start to dominate, leading to increased leakage currents [79]. Additionally, achieving low contact resistance and high carrier mobility in scaled devices is a significant challenge due to the Schottky barrier at the metal–semiconductor interface [80, 81].

In general, we think 2D vertical-channel transistors are more compatible and feasible for large-scale production.

Recent advancements in 2D transistor design have driven the miniaturization of the channel length, challenges posed by quantum effects, variability, heat dissipation, fabrication costs, and contact resistance need to be addressed to sustain this trend and realize the potential of smaller, more efficient electronic devices [18]. To tackle these issues, research is exploring novel contact schemes, such as the use of phase-engineered TMDs or the integration of high work function metals [82]. It should be noted that the channel width can also be scaled down to one atomic layer [75]. Additionally, the impact of environmental factors like ambient humidity on 2D TMD devices is an area of ongoing study.

### Reduction of the Gate Length

In a transistor, gate length—the distance over which an electric field controls current flow between the source and drain contacts—is crucial to its operation and overall device performance [83]. The gate length significantly influences the transistor's switching speed, power consumption, and drive current [54]. Shortening the gate length reduces the channel region traversed by carriers during operation, leading to quicker switching times. This enhances the transistor's operating speed, potentially improving the performance of the entire integrated circuit [84]. However, a shorter gate length can result in higher drive current due to the intensified electric fields in the channel. This increased current can lead to higher power consumption, presenting power management challenges in densely packed integrated circuits [85]. Despite these challenges, gate length reduction enables higher transistor density on chips, providing more computational power per unit area. This has propelled the extraordinary growth in processing power observed in the semiconductor industry, as exemplified by Moore's Law [25].

Nevertheless, shrinking the gate length into the nanometer regime brings several challenges. Quantum mechanical effects such as tunneling can become significant, increasing leakage currents and power dissipation [79]. When the gate length of silicon transistor decreases below 40 nm, adverse impacts such as drain-induced barrier lowering (DIBL) and self-heating effect (SHE) become more pronounced [86]. SHE can elevate device temperature, affecting reliability and lifespan, while DIBL can cause a significant shift in threshold voltage, increasing the device's off-state leakage current [54, 56].

The role of gate length becomes especially prominent in transistors based on 2D TMDs. 2D TMDs have demonstrated superior performance over traditional semiconductors like silicon in mitigating short-channel effects due to their thin bodies and large bandgaps [8]. The atomic thickness of 2D TMDs allows effective gate control even at extremely short gate lengths [39]. This permits aggressive device dimension scaling down to ~ nm level while maintaining effective electrostatic control over the channel, potentially leading to high-speed operation and low power consumption [87, 88].

Gate length also influences the contact resistance in 2D TMD transistors. As gate length decreases, the contact area reduces, potentially increasing contact resistance and lowering drive current [81]. Techniques such as contact engineering and the utilization of high-work-function metals have been proposed to address this issue [1].

Reducing a transistor’s gate length can significantly enhance performance and increase transistor density, it also introduces challenges related to quantum mechanical effects, power consumption, and device variability. Future research and innovation in transistor design and fabrication processes are necessary to overcome these challenges and continue the trend of transistor miniaturization. Ongoing advancements in 2D TMDs and further exploration of the interplay between gate length and device performance will contribute to the development of the next generation of miniaturized transistors.

As part of the exploration of the dimension and performance limits of transistors utilizing 2D TMDs, strategies for scaling down the gate length in traditional semiconductor transistors and those in 2D transistors will be compared to highlight the advantages of 2D TMDs.

#### Gate Length Reduction in Traditional Semiconductor Transistors

The reduction of gate length in transistors has been a key driver in advancing semiconductor technology. Various innovative techniques, each with its own advantages and challenges, have been adopted to achieve this. One significant technique is the transition from planar to three-dimensional transistor structures, including FinFET and Gate-All-Around (GAA) designs [56, 89]. These structures provide superior gate control and enable further gate length reduction without a significant increase in leakage current. The use of high-k dielectric materials in gate stacks is another innovative approach enabling gate length reduction. These materials offer higher gate capacitance, enhancing gate control at reduced gate lengths, minimizing leakage current, and improving device performance [90].

However, these methods present specific challenges. For example, fabricating 3D transistor structures is complex and necessitates advanced, technically challenging lithography techniques, such as EUV lithography. This can be costly. Additionally, using high-k dielectric materials can introduce interface traps and degrade carrier mobility, requiring further research and technological advancements to overcome these issues.

#### Gate Length Reduction in 2D Transistors

**Fig. 7:**
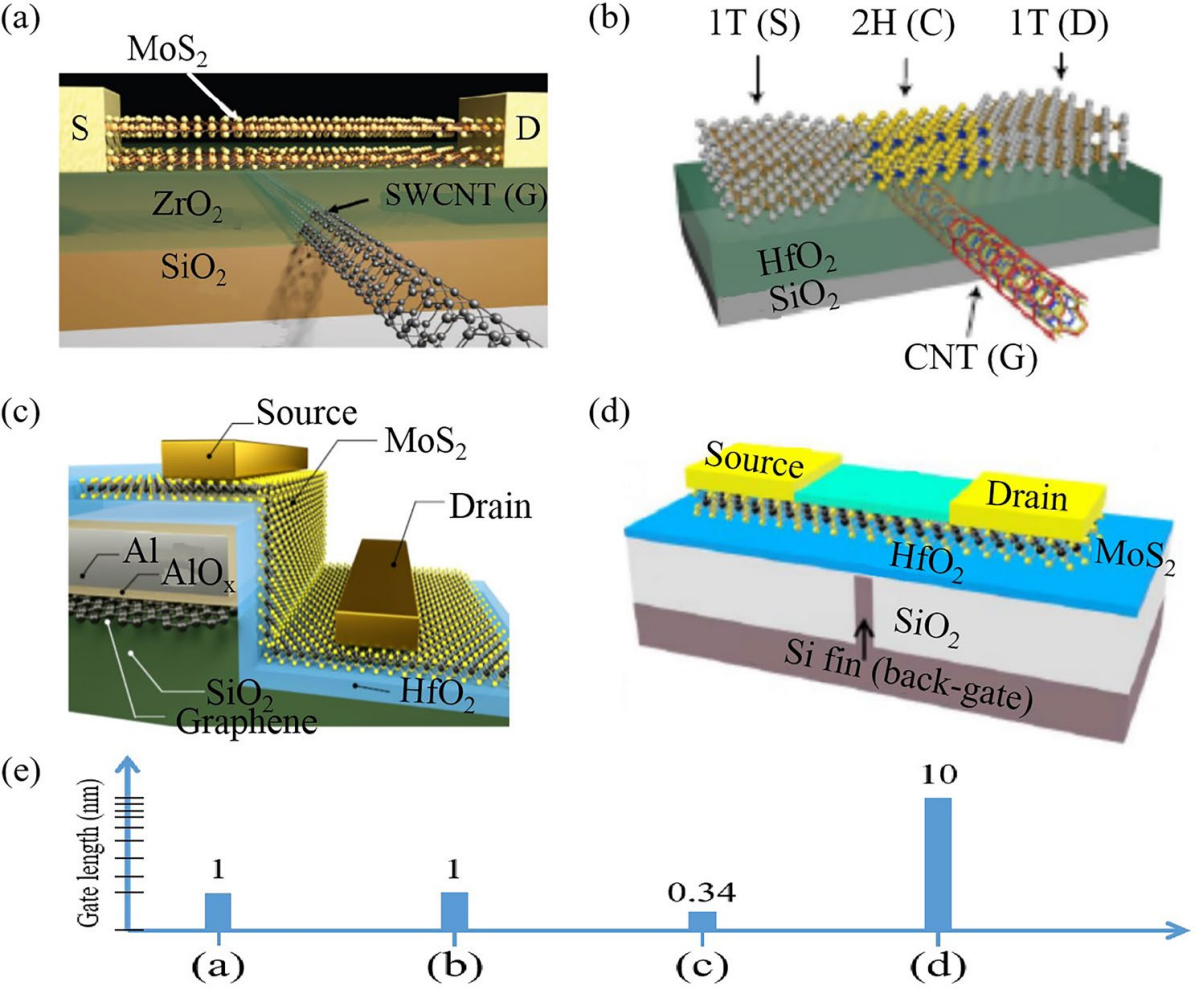
Scaling down the gate length of transistors. **a** MoS_2_ transistors with 1-nm gate lengths. Reproduced with permission. Reference [92] Copyright 2016, American Association for the Advancement of Science. **b** 1 T′/2H MoTe_2_ FET with a CNT gate. Reproduced with permission. Reference [93] Copyright 2019, Springer Nature. **c** Vertical MoS_2_ transistors with 0.34 nm monolayer graphene edge gate. Reproduced with permission. Reference [28] Copyright 2022, Springer Nature. **d** MoS_2_ Transistor with 10-nm Si fin gate length. Reproduced with permission. Reference [96] Copyright 2019, Institute of Electrical and Electronics Engineers Inc. **e** Gate lengths of above 2D transistors

In transistors based on 2D TMDs, gate length reduction has been a focal point for researchers aiming to push the boundaries of device miniaturization [8, 91]. Various innovative techniques have been explored (Fig. 7), each with their unique benefits and challenges. One significant advancement was the development of MoS_2_ transistors with a mere 1-nm gate length, employing a single-walled carbon nanotube [92] (Fig. 7a). The study demonstrated that MoS_2_ outperforms Si at sub-5-nm channel-length scaling limits. In another study, the single-walled carbon nanotube was used as the gate electrode in homojunction-channel (MoTe_2_) transistors, further confirming the potential for 1-nm gate length in 2D transistors [93] (Fig. 7b). The use of vertical MoS_2_ transistor structures, where the gate length is sub-1-nm, is another significant technique [28] (Fig. 7c). In this work, Fan Wu et al. showed side-wall MoS_2_ transistors with a single graphene layer edge as the gate electrode, achieving a physical gate length of sub-1 nm. These devices demonstrated subthreshold swing values down to 117 mV dec^−1^ and on/off ratios up to 1.02 × 10^5^, paving the way for continued transistor scaling in line with Moore’s law [28]. Dynamically doped transistors offer another innovative approach [94]. The 2021 study introduced the concept of a doping gate length (LDG), which is longer than traditional gate length (L) but does not necessitate a larger contact gate pitch (CGP) footprint. This technique can reduce gate length without increasing the overall transistor size, meeting the 2031 International Roadmap for Devices and Systems (IRDS) dimensional objectives for the so-called 1-nm-technology node and beyond [94]. Furthermore, the ab initio quantum-transport methods have successfully simulated sub-5 nm gate-length monolayer MoS_2_ transistors [95].

In another study, Yu Pan et al. investigated MoS_2_ transistors with a 10-nm gate length, using a Si Fin structure as the gate electrode [96, 97] (Fig. 7d). This novel design achieved an on/off ratio of over 10^6^, showing promise for future scaled 2-D material transistors. Figure 7e shows the quantitative comparation of gate lengths.

Nonetheless, these techniques present challenges. Gate length reduction to the nanometer scale can lead to increased leakage current, a significant challenge for device performance and energy efficiency. Moreover, the fabrication processes for creating such small structures are complex and require high precision, which can be challenging to achieve and scale for mass production. Advancements in material science and device engineering are expected to pave the way for further miniaturization of these devices [60, 98].

### Minimization of the Source/Drain Contact Length

The contact length, the distance at which the source and drain regions establish contact with the channel, profoundly influences a transistor's resistance, capacitance, and consequently, its speed and power consumption [83]. This parameter is particularly significant in transistors based on 2D TMDs, where it directly impacts performance and energy efficiency [99].

In transistor operation, carriers traverse between the channel and contact metals across an effective pass length, known as the transfer length. According to the current crowding model, carriers preferentially enter the semiconductor via the periphery of metal–semiconductor contact regions, rendering the transfer length considerably smaller than the contact length [100]. Thus, the theory proposes that the contact length can continuously scale to the transfer length, providing a theoretical basis for contact length minimization. From an energy efficiency perspective, reducing the contact length decreases the voltage drop across the source and drain regions, thereby lowering power consumption during transistor operation [101]. Additionally, a smaller contact length can decrease gate capacitance, potentially resulting in a lower threshold voltage and reduced power consumption [8]. This is particularly relevant in modern integrated circuits, where power consumption and dissipation are pivotal considerations.

However, reducing the contact length also diminishes the contact area, potentially increasing contact resistance. Elevated contact resistance can reduce the drive current, potentially impairing device performance, including switching speed [54]. The contact length also impacts short-channel effects [56]. A diminished contact length can augment short-channel effects, such as drain-induced barrier lowering (DIBL) and velocity saturation, adversely affecting device performance. Nevertheless, inherent properties of 2D TMDs, like their thin body and large bandgap, can help counteract these effects [102, 103]. Despite the benefits, minimizing the source/drain contact length presents challenges. As contact length decreases, maintaining effective electrical contact between the source/drain regions and the channel grows increasingly difficult [104, 105]. Increased contact resistance can negate the benefits of reduced series resistance and may induce variability in device performance. Furthermore, as the contact length shrinks to nanometer scales, the fabrication process becomes more intricate. Advanced lithography and self-aligned techniques are often necessary, introducing additional cost and complexity.

Contact length in 2D TMD transistors significantly influences device energy efficiency and performance. However, the net impact is a complex interplay of various factors, including contact resistance, parasitic capacitance, gate capacitance, and short-channel effects. Therefore, careful optimization of the contact length is crucial to maximize the performance and energy efficiency of these devices.

As part of the exploration of the dimension and performance limits of transistors utilizing 2D TMDs, strategies for scaling down the contact length in traditional semiconductor transistors and those in 2D transistors will be compared to highlight the advantages of 2D TMDs.

#### Source/Drain Minimization in Traditional Semiconductor Transistors

The minimization of source/drain contact length constitutes a key aspect of the MOSFET scaling trend. Various techniques and technological advancements have been utilized to reduce contact length, each bearing its own efficacy and challenges. The implementation of Raised Source/Drain (RSD) structures has emerged as an effective strategy [106]. RSDs decrease contact length by forming additional silicon layers atop the source and drain regions, thereby reducing the series resistance and enhancing transistor performance. The introduction of Self-Aligned Contact (SAC) techniques constitutes another significant development [107]. SAC processes align contacts directly over the transistor's active regions, enhancing precision while reducing contact length. The use of silicide, a silicon-metal compound, has also proven instrumental in diminishing contact length [108]. Silicide reduces contact resistance between the metal contact and the silicon of the source/drain regions, facilitating further contact length reduction.

Despite their benefits, these methods each pose unique challenges. The fabrication of RSD structures necessitates precise epitaxial growth techniques, which are technically demanding and costly. SAC techniques, despite facilitating excellent alignment, require intricate lithography and etching processes. The use of silicide can introduce issues such as agglomeration and junction spiking, potentially degrading transistor performance. Moreover, as contact lengths continue to shrink, maintaining reliable, low-resistance contacts becomes increasingly challenging. Continued research and innovation are required to sustain the trend of contact length reduction, fostering more efficient and compact transistors.

#### Source/Drain Minimization in 2D Transistors

**Fig. 8:**
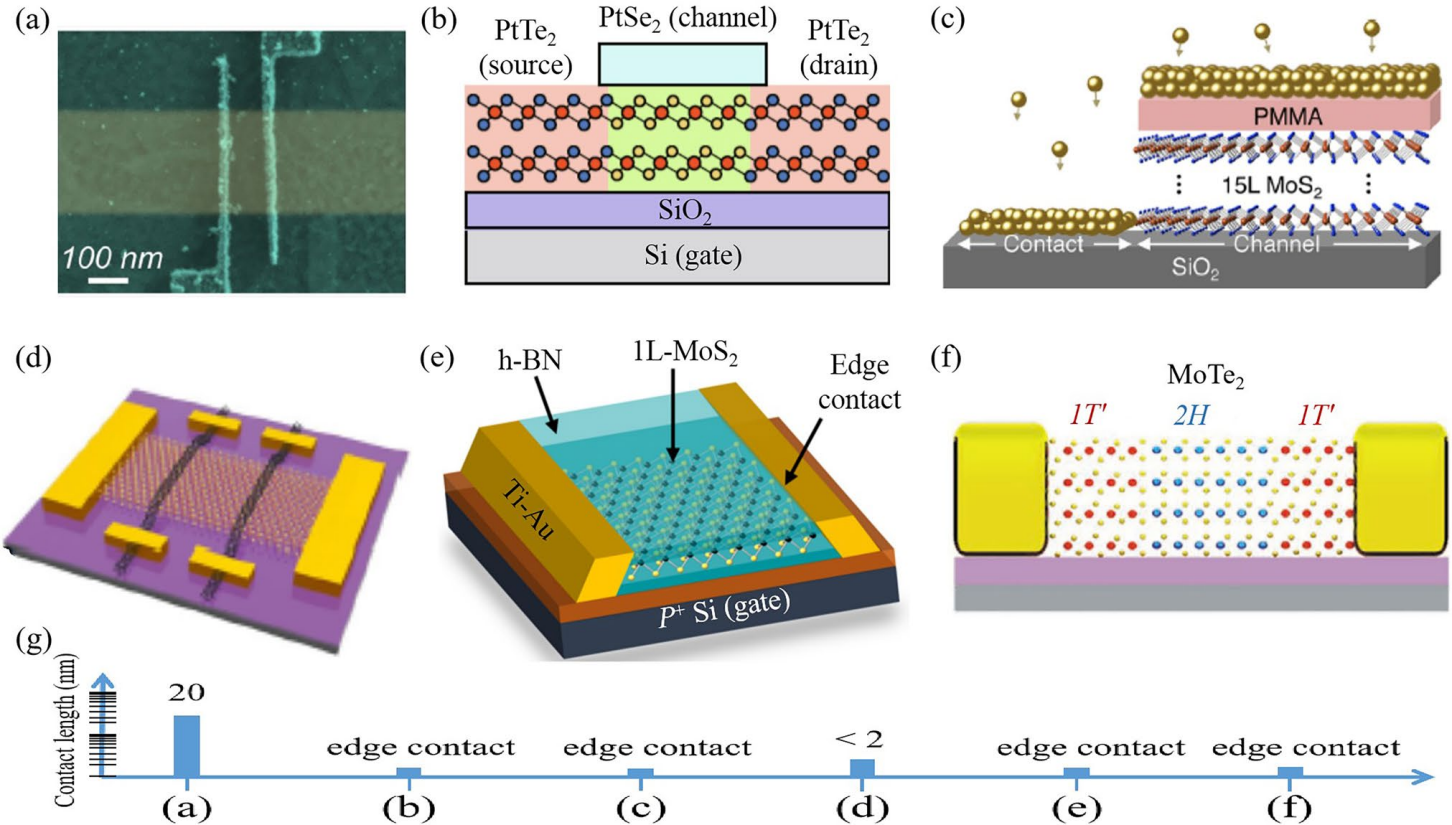
Scaling down the contact length of transistors. **a** Ultrascaled contacts for monolayer MoS_2_ FET. Reproduced with permission. Reference [109] Copyright 2023, American Chemical Society. **b** PtSe_2_ FET with PtTe_2_ edge contacts. Reproduced with permission. Reference [111] Copyright 2022, Elsevier. **c** MoS_2_ transistors using in situ edge contacts. Reproduced with permission. Reference [112] Copyright 2019, American Chemical Society. **d** MoS_2_ FET with single-walled-carbon-nanotube contacts. Reproduced with permission. Reference [29] Copyright 2023, Springer Nature. **e** MoS_2_ FET with one-dimensional edge contacts. Reproduced with permission. Reference [113] Copyright 2019, American Chemical Society. **f** MoTe_2_ FET with phase-transition contacts. Reproduced with permission. Reference [116] Copyright 2020, Wiley–VCH Verlag. **g** Contact lengths of above 2D transistors

The reduction of contact length in 2D TMD transistors is a vital aspect of device miniaturization. Several methods have been proposed and implemented (Fig. 8), each with its unique efficacy and challenges. Advanced lithography techniques, such as EUV lithography and electron-beam lithography, are primary methods for reducing contact length [31, 109, 110] (Fig. 8a). These techniques afford precise control over the patterning process, enabling the fabrication of devices with nanoscale contact lengths. However, they tend to incur high equipment costs and complex processing steps, which can hinder mass production. The use of metallic edge contacts has proven effective. Edge-contact technique has been employed for PtSe_2_ and MoS_2_ transistors [111–113] (Fig. 8b, c, e). These edge-contact transistors exhibit contact performance comparable to top/bottom contact configurations but with a significantly reduced footprint. Similar metallic edge contacts have also been shown in graphene-nanoribbon [114] and graphene transistors [115]. However, this fabrication process involves complex steps. Phase engineering also can be used for edge contacts in 2D TMD transistors [116] (Fig. 8f). Another innovative technique involves the use of one-dimensional single-walled carbon nanotube electrodes as the source/drain electrodes of 2D transistors [29] (Fig. 8d). Figure 8g shows the quantitative comparation of contact lengths. This allows the contact length to scale into the sub-2 nm region, providing a novel approach for future nanoelectronics miniaturization. Semimetal graphene-nanoribbons have also been used for the source/drain electrodes of 2D transistors, exhibiting excellent device performance [117]. However, these techniques necessitate precise alignment processes, escalating the fabrication difficulty [118].

In general, we think phase-transition-contact transistors are more compatible and feasible for large-scale production.

Various methods have been developed for source/drain contact length reduction in 2D TMD transistors, each carrying unique challenges that need to be addressed. Ongoing advancements in material science and device engineering are anticipated to facilitate further reductions in source/drain contact length.

### Reduction of the Dielectric Thickness

The dielectric thickness, often associated with gate oxide thickness in a MOSFET, significantly influences transistor operation. It directly affects the transistor's performance, power consumption, and leakage current [119], particularly relevant in the context of transistors based on 2D TMDs [120].

In a FET, the dielectric material separates the gate electrode from the conductive channel, typically a 2D TMD material like MoS_2_. The thickness of this dielectric layer, often termed gate oxide thickness, directly impacts the electrostatic control of the gate over the channel [121]. A decrease in dielectric thickness enhances gate control, improving transistor performance parameters such as on-current and subthreshold swing. Hence, dielectric thickness reduction remains a key aspect of transistor scaling. However, a decrease in dielectric thickness also leads to an increase in leakage current, presenting a significant challenge in transistor scaling. Two primary mechanisms of leakage current in a MOSFET are gate oxide leakage [122] and subthreshold leakage [123]. Gate oxide leakage occurs when electrons tunnel through the thin dielectric layer, a phenomenon known as direct tunneling. As the dielectric thickness decreases, direct tunneling probability increases, resulting in a higher gate leakage current. Apart from power wastage, leakage current can induce issues such as heating, noise, and even device failure [124]. Subthreshold leakage, in contrast, arises from the finite off-state current in the transistor when it should be in the ‘off’ state. This leakage is exacerbated by dielectric thickness reduction because it leads to a decrease in the transistor's threshold voltage.

Several strategies have been proposed to balance dielectric thickness and leakage current in 2D TMD transistors. These include the use of high-k dielectrics, offering high permittivity while minimizing leakage current [36, 87], and the implementation of passivation techniques to reduce trap densities and surface roughness [125]. Moreover, innovative device architectures, such as double-gate or gate-all-around structures, can enhance gate control while maintaining a relatively thick dielectric layer [126, 127].

The dielectric thickness is a crucial parameter in 2D TMD transistor operation, affecting both the transistor's performance and leakage current. Despite the challenges, ongoing research is exploring new materials and device architectures to optimize this critical parameter [18], continuing the trend of transistor scaling. Reducing the dielectric thickness has been a principal strategy for enhancing the performance of MOSFETs.

#### Reduction of the Dielectric Thickness in Traditional Semiconductor Transistors

Several strategies have been employed to Traditional Semiconductor Transistors, each with its respective effectiveness and challenges. An early strategy entailed thinning the silicon dioxide (SiO_2_) layer used as the gate dielectric [56]. While this approach succeeded in improving device performance by enhancing gate control over the channel region, quantum mechanical tunneling-induced leakage currents significantly increased as the SiO_2_ layer was reduced to near-atomic scales [119]. To mitigate this issue, high-k dielectric materials were introduced as alternatives to SiO_2_ [90]. High-k materials, such as Hafnium Oxide (HfO_2_), possess a higher dielectric constant (k), which allows for a physically thicker layer offering the same capacitive properties as a thinner SiO_2_ layer, thereby reducing leakage current. However, these materials present challenges such as increased gate leakage due to defects and traps within the high-k material, and threshold voltage instability [128]. Another strategy involves the utilization of multi-gate transistor architectures, like FinFETs [107]. These structures use a three-dimensional "fin" shaped channel region, surrounded by the gate on multiple sides, allowing superior gate control over the channel even with a thicker dielectric layer, thereby reducing leakage current. However, FinFET structures are more complex to fabricate and integrate into existing manufacturing processes. While strategies such as the use of high-k materials and multi-gate architectures have proved effective in achieving dielectric thickness reduction, they introduce new challenges. Further research and innovation are required to address these challenges and perpetuate the trend of transistor scaling.

#### Reduction of the Dielectric Thickness in 2D Transistors

**Fig. 9:**
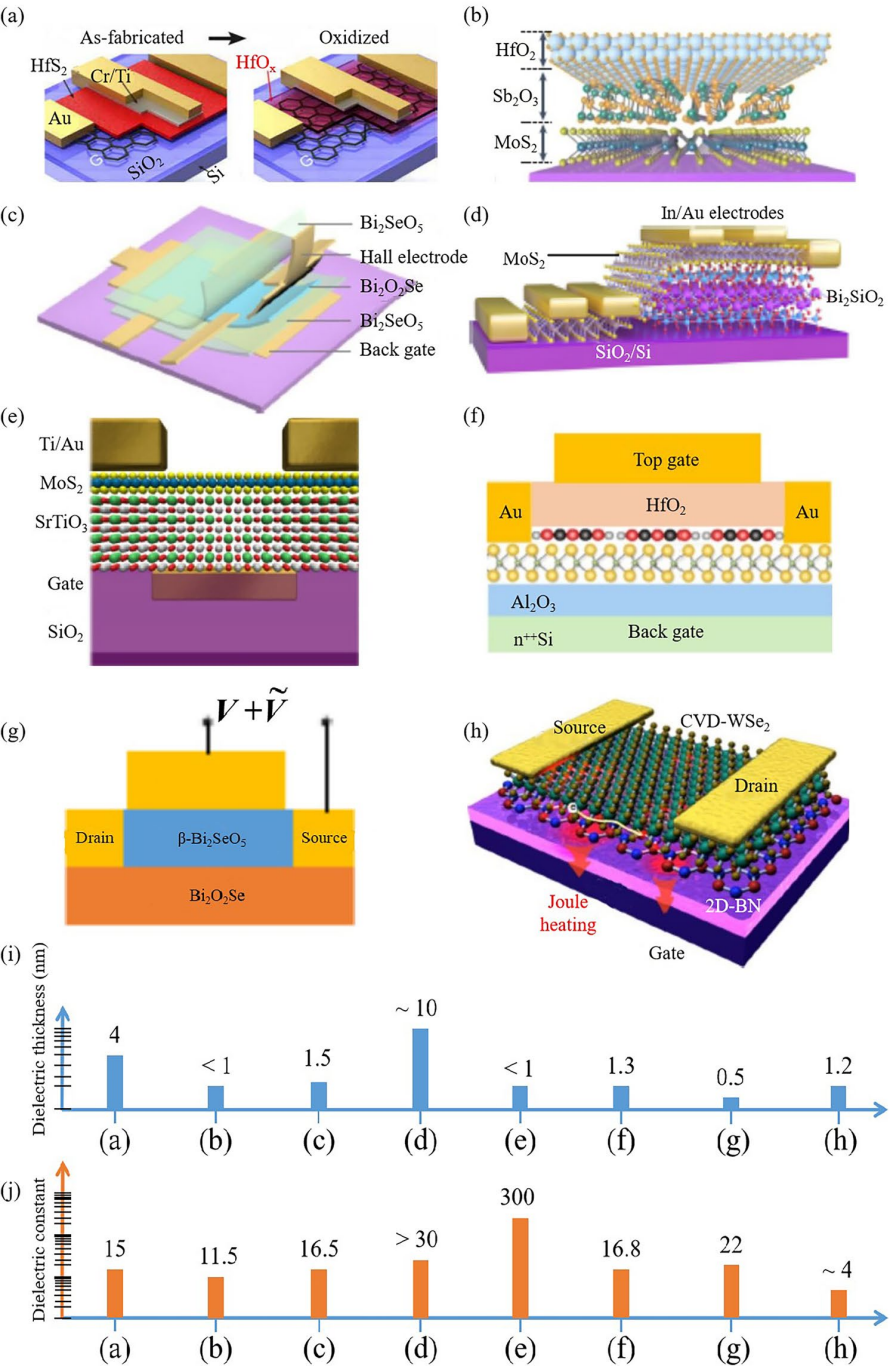
Scaling down the dielectric thickness of transistors. **a** Laser-writable high-k dielectric for van der Waals nanoelectronics. Reproduced with permission. Reference [129] Copyright 2019, American Association for the Advancement of Science. **b** The hybrid HfO_2_/Sb_2_O_3_ dielectrics integrated on 2D MoS_2_. Reproduced with permission. Reference [130] Copyright 2023, Springer Nature. **c** Bi_2_O_2_Se transistor with Bi_2_SeO_5_ dielectric nanosheets. Reproduced with permission. Reference [131] Copyright 2023, Springer Nature. **d** Back-gate MoS_2_ four-probe FETs device. Reproduced with permission. Reference [132] Copyright 2023, Springer Nature. **e** MoS_2_ back-gated FET with SrTiO_3_ dielectric material. Reproduced with permission. Reference [30] Copyright 2022, Springer Nature. **f** MoS_2_ FET with hybrid PTCDA/HfO_2_ gate stack. Reproduced with permission. Reference [133] Copyright 2019, Springer Nature. **g** Bi_2_O_2_Se transistor with Bi_2_SeO_5_ dielectric nanosheets. Reproduced with permission. Reference [134] Copyright 2022, Springer Nature. **h** WSe_2_ FET using 2D-BN dielectric interface. Reproduced with permission. Reference [150] Copyright 2019, Springer Nature. **i** Dielectric thickness of above 2D transistors. **j** Dielectric constants of above 2D transistors

In 2D TMD transistors, reducing dielectric thickness is also a strategic approach to enhance the electrostatic control of the gate over the channel, thereby improving transistor performance. Below, we analyze some strategies for dielectric thickness reduction (Fig. 9), their effectiveness, and associated challenges. One is the application of high-k dielectrics. High-k dielectric materials, such as Hf(Zr)_1+x_O_2_, Sb_2_O_3_, SrTiO_3_, Bi_2_SiO_5_, LaOCl, Sr_2_Nb_3_O_10_, fluoride film offer high permittivity, allowing for thinner effective oxide thickness while maintaining relatively low leakage current [30, 129–145] (Fig. 9a–h). Figure 9i, j show the quantitative comparation of dielectric thicknesses and constants, respectively. These materials, having been successfully integrated into 2D TMD transistors, result in improved subthreshold swing and on-current. However, it is difficult to acquire clean interface between the high-k dielectric and the TMD channel [121, 146]. Passivation technique is another approach to reduce dielectric thickness. Surface passivation techniques, including chemical and physical approaches, can reduce trap densities and surface roughness in 2D TMD materials [147–149], thereby reducing the leakage current and enabling the use of thinner dielectrics. However, these techniques often demand high-temperature processes potentially damaging the 2D TMD layers. Another approach is to use novel device architectures. For instance, double-gate or gate-all-around structures can provide improved gate control with a relatively thick dielectric layer [35, 126]. These architectures can reduce the electric field at the gate oxide/TMD interface, thereby reducing leakage current. However, the fabrication of these structures is more complex and can increase the overall device cost. The utilization of 2D materials as dielectrics also can effectively reduce dielectric thickness. 2D Hexagonal Boron Nitride (h-BN) has been proposed as a dielectric material for 2D TMD transistors [150]. These materials can be scaled down to a few atomic layers, allowing for ultra-thin dielectrics with low leakage current. However, integrating 2D h-BN as dielectrics presents challenges such as material synthesis, device fabrication, and interface quality.

In general, we think dielectric materials such as Sb_2_O_3_, Bi_2_SeO_5_, SrTiO_3_, and HfO_2_, hold the promise for scalable production. The corresponding high-k dielectric transistors are more compatible and feasible for large-scale production.

Strategies for dielectric thickness reduction in 2D TMD transistors involve a trade-off between improved transistor performance and increased leakage current. Despite these challenges, ongoing research continues to explore new materials, techniques, and architectures that can optimize dielectric thickness while maintaining low leakage current.

## Performance of 2D TMDs Transistors

In the realm of semiconductor technology, the relentless push towards miniaturization coupled with the quest for higher performance and energy efficiency has led to the exploration of two-dimensional (2D) materials for transistors. These materials, often only a few atoms thick, promise a revolutionary step beyond the limitations of traditional silicon-based devices. However, to fully harness the potential of 2D transistors, several key electrical characteristics must be optimized. These include reducing source/drain contact resistance, minimizing subthreshold swing, decreasing hysteresis loop, enhancing carrier mobility, and improving the on/off current ratio. Optimizing these electrical characteristics in 2D transistors is not just about pushing the frontiers of miniaturization; it is about achieving greater computational power, energy efficiency, and reliability in the electronics that permeate every aspect of modern life, from smartphones to supercomputers. The continued advancement in 2D transistor technology promises to be a cornerstone in the evolution of next-generation electronics.

### Reduction of Source/Drain Contact Resistance

The source/drain (S/D) contact resistance in a transistor significantly affects overall device performance and power consumption, especially in transistors based on 2D TMDs [151]. The contact resistance largely determines the effective mobility of charge carriers, regulating the current flow rate through the transistor, and thereby impacting the overall device performance [152]. Elevated S/D contact resistance can considerably degrade transistor performance by reducing the device's current drive capability [153]. The contact resistance forms a barrier that hinders carrier flow from the source to the channel and from the channel to the drain, thereby limiting the transistor's switching speed and affecting the overall device performance [56]. This issue is especially problematic for high-frequency applications that demand fast switching times for efficient operation [151]. Furthermore, high S/D contact resistance can augment power consumption. In a transistor, power is consumed not only during the switching operation but also when a current flows through the device due to encountered resistance. Consequently, high contact resistance can lead to increased power dissipation, reducing energy efficiency and potentially causing thermal management problems in high-performance electronic systems. This issue is of particular concern in the current era of electronic devices where energy efficiency is paramount. For 2D TMD-based transistors, the thin nature of these 2D materials suggests that the contact region has a larger impact on overall device performance compared to bulkier three-dimensional materials [81, 154–158].

Therefore, to enhance the performance of these transistors, it is essential to reduce the contact resistance between the source/drain and the 2D TMD material. This reduction can be achieved through various methods, such as implementing novel contact materials [31, 159], interface engineering [152, 160], contact engineering techniques [80, 161, 162], self-aligned contact processes [151], or by modifying the 2D TMD material itself [163, 164]. However, these strategies can introduce additional challenges, including increased fabrication complexity, potential degradation of the 2D TMD material, and potential incompatibility with existing semiconductor fabrication processes [157]. The contact engineering techniques are typically employed to reduce contact resistance. Yet, in the context of 2D TMDs, the ultrathin nature of these materials presents unique challenges for contact engineering. Traditional methods used in silicon-based electronics, such as selective ion implantation, are not applicable due to the ultrathin body of monolayer and few-layer TMDs [165]. Furthermore, the variation in electron affinity, band gap, and band alignments among different TMDs complicates the contact engineering process [157]. Thus, additional research is needed to develop effective strategies for reducing contact resistance that do not compromise the intrinsic properties of the 2D TMD material and that are compatible with existing processes [166].

#### Current Strategies in Resistance Reduction in Traditional Semiconductor Transistors

FETs are the bedrock of contemporary semiconductor devices. Their performance is profoundly influenced by the source/drain contact resistance. The current strategies employed to reduce this contact resistance are multifarious, each with distinct levels of efficacy and unique challenges.

A prevalent strategy involves utilizing metal silicide contacts such as nickel silicide or titanium silicide. These materials form a low-resistance ohmic contact with silicon, thereby enhancing device performance [167]. However, the formation of these silicides necessitates high-temperature annealing, potentially leading to unwanted diffusion or junction leakage [19]. Another approach involves heavily doping the source/drain regions to diminish the Schottky barrier height, thereby reducing contact resistance. Nevertheless, heavy doping can result in increased leakage current, consequently degrading transistor performance [168].

Two strategies exist for reducing source/drain contact resistance in Si FETs, each carries its own set of challenges. Therefore, current research continues to probe novel materials and innovative fabrication techniques to further optimize the performance of Si FETs [169].

#### Current Strategies in Resistance Reduction in 2D Transistors

**Fig. 10:**
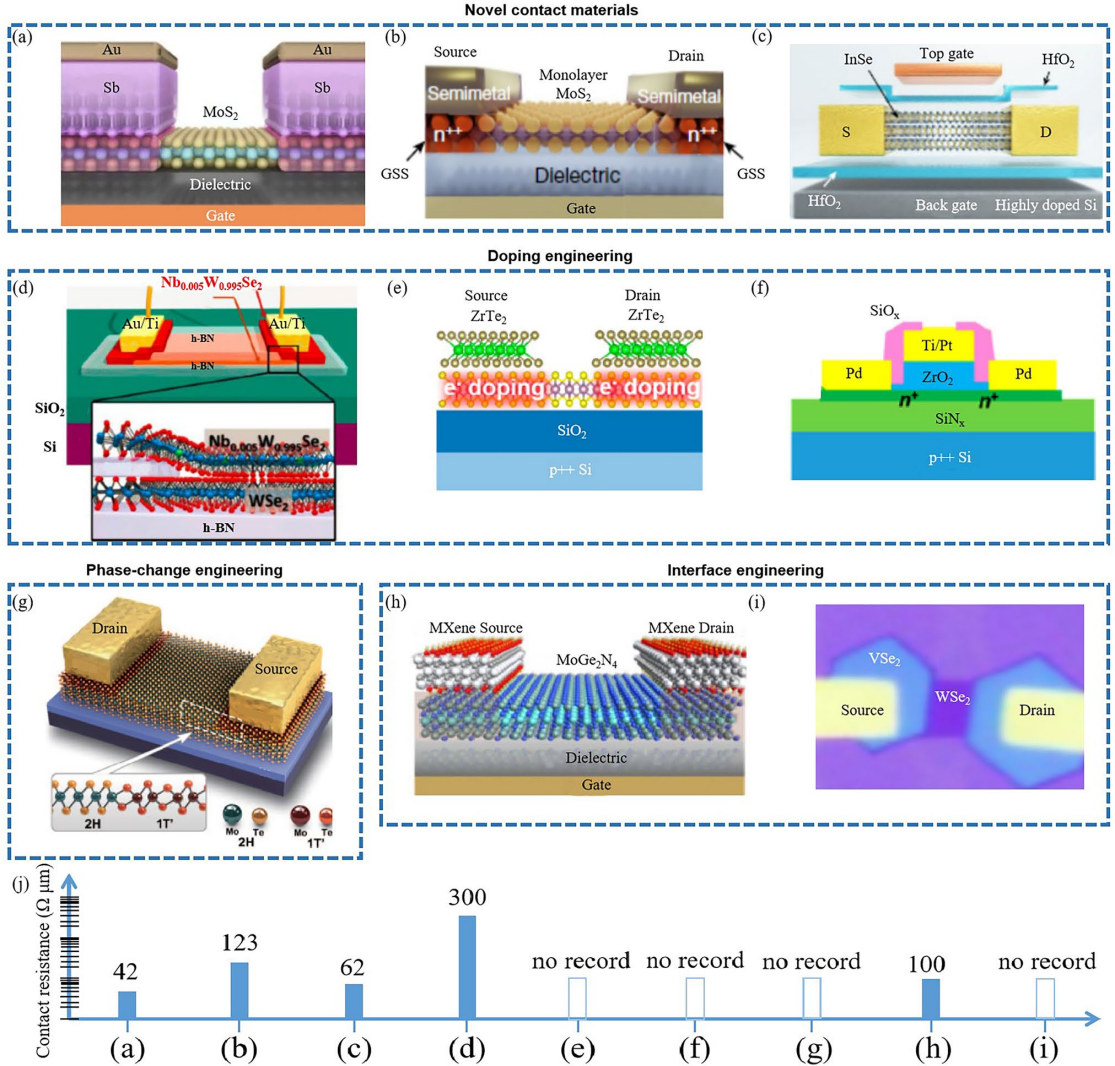
Contact resistance reduction of 2D transistors involves four strategies such as novel contact materials, doping engineering, interface engineering, and phase-change engineering. **a** The quantum limit in MoS_2_ FET. Reproduced with permission. Reference [31] Copyright 2023, Springer Nature. **b** MoS_2_ FET with semimetal (Bi) contacts. Reproduced with permission. Reference [156] Copyright 2021, Springer Nature. **c** Double-gate InSe FET. Reproduced with permission. Reference [171] Copyright 2023, Springer Nature. **d** WSe_2_ FET with degenerately p-doped WSe_2_ (Nb_0.005_W_0.995_Se_2_) contacts. Reproduced with permission. Reference [172] Copyright 2016, American Chemical Society. **e** ZrTe_2_-contacted MoS_2_ transistor. Reproduced with permission. Reference [160] Copyright 2023, American Chemical Society. **f** Ideal spacer doping layer for 2D devices. Reproduced with permission. Reference [164] Copyright 2023, American Chemical Society. **g** MoTe_2_ device with a 1 T'/2H phase homojunction. Reproduced with permission. Reference [173] Copyright 2015, American Association for the Advancement of Science. **h** 2D MoGe_2_N_4_ FET with Mxene contacts. Reproduced with permission. Reference [174] Copyright 2023, Royal Society of Chemistry. **i** WSe_2_ FET with VSe_2_ contact. Reproduced with permission. Reference [152] Copyright 2023, Springer Nature. **j** Contact resistance of above 2D transistors

The reduction of source/drain (S/D) contact resistance is a pivotal aspect in 2D transistor design and fabrication, significantly influencing overall device performance [21, 170]. Various strategies have been devised to address this challenge (Fig. 10), each presenting unique benefits and drawbacks.

One approach involves using novel contact materials. Antimony (Sb) or Bismuth (Bi) or Yttrium (Y) have been employed to reduce contact resistance in n-type transistors [31, 156] (Fig. 10a, b). Sb-MoS_2_ contact can approach the quantum limit due to hybridization of M–S energy bands at the Fermi energy [31]. Bi-MoS_2_ contact is ohmic contact, owing to the suppression of metal-induced gap states [156]. Y-InSe contact realize the ohmic contact, benefiting from that Y doping converts semiconducting InSe into semimetallic Y-InSe [171] (Fig. 10c). While these materials can effectively decrease contact resistance, they can introduce complications including potential reactions with semiconductor materials, stability issues, and integration challenges [175]. Interface engineering is another promising strategy. This approach entails creating an ultra-thin interfacial layer between the metal and the semiconductor to ensure ohmic contact, aiding in reducing contact resistance [152, 160, 171–173, 176–180] (Fig. 10d, e, g, i). However, maintaining precise control of the interfacial layer properties, such as thickness, uniformity, and chemical composition, can be technically challenging. The implementation of self-aligned contact processes is a third approach. Here, the S/D regions are formed after defining the gate stack, thereby allowing the contacts to be closely aligned with the gate, reducing parasitic resistance [151]. However, this technique demands a high level of process control and can introduce complexity into the fabrication process. Doping strategies have also been used to reduce contact resistance. For instance, implanting dopants into the S/D regions can augment the carrier concentration, thereby reducing the contact resistance [163, 164, 181, 182] (Fig. 10f). However, this approach can induce defects and degrade the intrinsic properties of the semiconductor material. Contact engineering techniques, such as edge contact formation [82], local pressurization contact [161], van der Waals clean interface contacts [154, 159, 183–191], phase engineering [192–195], and superplastic deformation [162], have been effective in reducing contact resistance. Figure 10j shows the quantitative comparation of contact resistance. These strategies involve creating contacts at the edge of the 2D TMD, instead of the top surface, which has been shown to reduce contact resistance due to the higher density of states at the edges of these materials. However, while promising, contact engineering techniques can increase fabrication complexity and may not be feasible for all device architectures.

In general, we think novel-contact-material transistors are more compatible and feasible for large-scale production.

Several strategies for reducing contact resistance in 2D TMD transistors are effective, each carries its own set of challenges. Additional research is needed to develop, optimize, and overcome the challenges associated with these strategies, ensuring compatibility with existing fabrication processes without compromising the inherent advantages of 2D TMDs [166, 174] (Fig. 10h). Recently, machine learning has been employed to screen low-contact electrode for 2D semiconductor [196], indicating its prospect in resistance reduction in 2D transistors.

### Reduction of Subthreshold Swing

The subthreshold swing (SS) is a critical parameter in transistor operation, quantifying the required change in gate voltage to achieve an order of magnitude change in the drain current within the subthreshold region. Essentially, the SS characterizes the transistor's "off" state efficiency [123]. Notably, SS impacts power efficiency. Despite a transistor being in its "off" state, a small amount of leakage current persists. The magnitude of this leakage current is contingent upon the SS. A larger SS correlates with more leakage current, thereby escalating static power consumption [197]. This becomes particularly detrimental in low-power applications, like mobile devices and wearable technology, where power efficiency is paramount [198]. Hence, a lower SS enhances the transistor's switching efficiency between the "on" and "off" states, subsequently reducing power consumption.

Reducing the SS can amplify both the speed and power efficiency of a transistor. However, due to thermal constraints, the SS of conventional MOSFETs cannot fall below a certain threshold (approximately 60 mV decade^−1^ at room temperature) [199]. To surpass this limit, innovative transistor designs and technologies are under exploration. For instance, Tunnel Field-Effect Transistors (TFETs) can achieve an SS beneath the thermal limit by utilizing band-to-band tunneling [200]. Nonetheless, TFETs encounter challenges, including diminished on-currents and complexities in fabricating high-quality tunneling junctions [201].

An alternative approach involves Negative Capacitance Field-Effect Transistors (NCFETs), which use ferroelectric materials in the gate stack to provide internal voltage amplification and diminish the SS [202]. However, the practical implementation of NCFETs confronts obstacles such as ferroelectric material integration and reliability [203].

2D monolayer TMDs, such as MoS_2_, WS_2_, and WSe_2_, possess distinctive properties like a direct bandgap and high carrier mobility, positioning them as promising candidates for next-generation electronic devices. Regarding transistors based on 2D TMDs, the SS assumes considerable significance due to these materials' atomic thickness. Unlike bulk semiconductors, 2D TMDs can potentially attain an ideal SS close to the thermal limit of 60 mV dec^−1^ at room temperature, thanks to their ultra-thin body that ensures robust gate control and an efficient suppression of the off-state current [39]. A low SS is advantageous for realizing high-precision, low-power digital circuits. It is especially important in low-power applications, where a substantial part of the power is consumed while the transistor is in the subthreshold region [204]. Therefore, the potential of 2D TMDs to achieve a low SS is a crucial benefit in the pursuit of power-efficient electronics.

Several strategies have recently been proposed to address these issues, including negative-capacitance FETs [205], tunneling FETs [206], impact ionization FETs [207], resistive gate FETs [208], and Dirac-source FET [209]. However, achieving a low SS in 2D TMD transistor devices is fraught with challenges. Material defects, interface traps, and contact resistance can all degrade the SS. These issues can be exacerbated by the inherent sensitivity of these atomically thin materials to their environment [18].

The role of SS in transistor performance is crucial, particularly regarding power efficiency. Transistors based on 2D TMDs show promise in achieving low SS values, potentially leading to more power-efficient devices. However, further research is needed to overcome the challenges associated with these materials. Therefore, meticulous optimization of the device structure and material quality is mandatory to harness the potential benefits of 2D TMDs in achieving low SS and high-power efficiency.

#### Current Strategies in Subthreshold Swing Reduction in Traditional Semiconductor Transistors

Subthreshold swing (SS) reduction has emerged as a pertinent research focus in transistor technology, with various promising strategies under exploration. Tunnel Field-Effect Transistors (TFETs) represent a compelling approach. They exploit band-to-band tunneling to activate the transistor, achieving an SS lower than the thermal limit of 60 mV decade^−1^ at room temperature [79]. However, TFETs often exhibit low on-currents, and fabricating high-quality tunneling junctions remains a formidable task [201]. Negative Capacitance Field-Effect Transistors (NCFETs) offer another promising avenue. By incorporating ferroelectric material into the gate stack, NCFETs achieve internal voltage amplification, thereby reducing the SS [202]. Nonetheless, integrating ferroelectric materials into transistor structure poses challenges, and reliability concerns linked to these materials persist [203]. Nanoelectromechanical FETs (NEM-FETs) present a potential method for lowering SS beneath the thermal limit [210]. Upon applying the threshold voltage to the transistor's gate electrode, an abrupt mechanical movement brings the electrode close to the gate dielectric layer. This movement induces rapid carrier increase in the channel, facilitating swift off-to-on transition, resulting in a significantly low SS. However, fabricating a suspended gate in a MOSFET proves challenging, necessitating advanced techniques and increasing process variability.

Various strategies for SS reduction and consequent transistor performance improvement exist, each presents unique challenges. Continuous research and innovation are imperative for refining these strategies and devising novel approaches.

#### Current Strategies in Subthreshold Swing Reduction in 2D Transistors

**Fig. 11:**
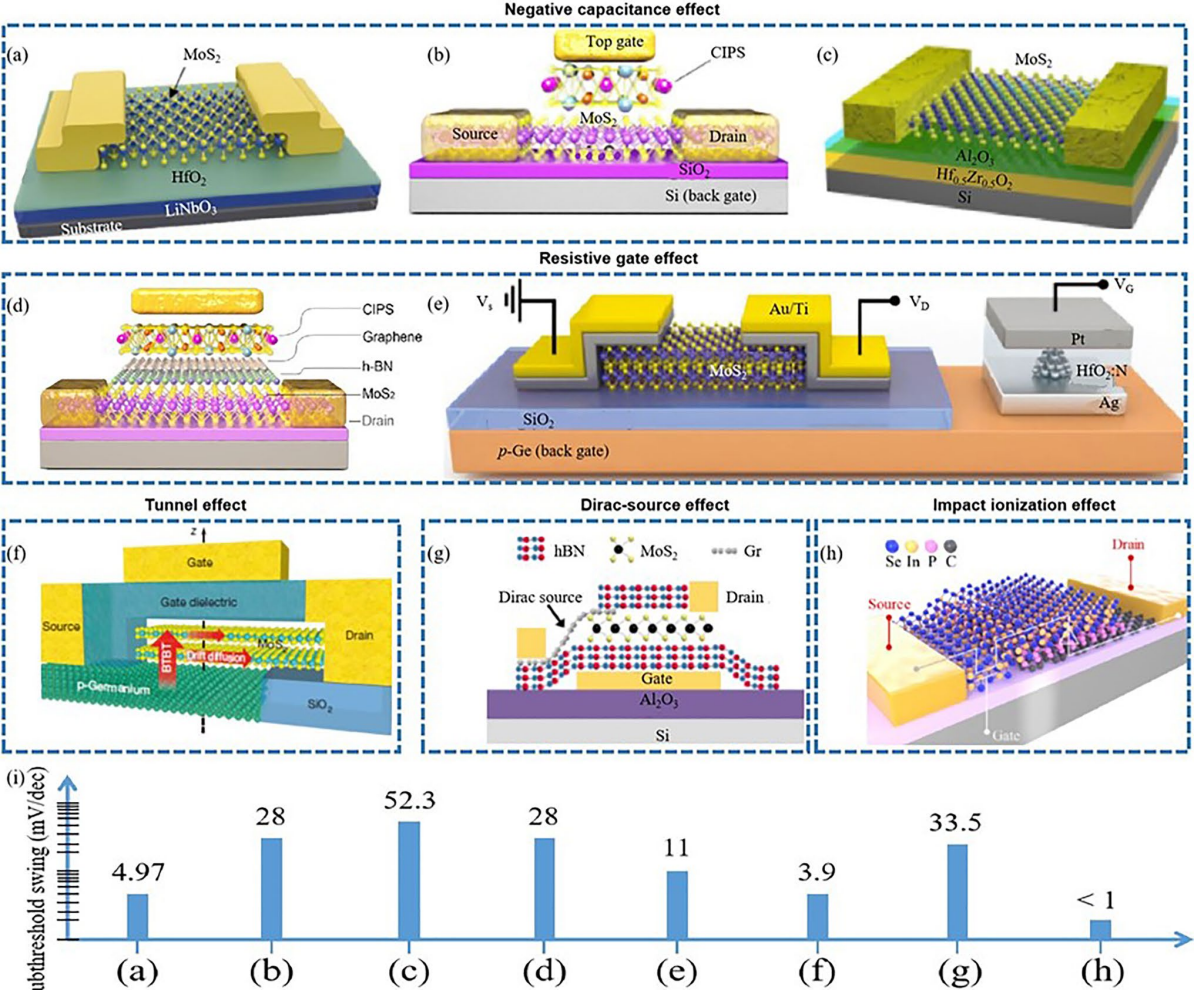
Subthreshold swing reduction of 2D transistors involves negative capacitance effect, resistive gate effect, tunnel effect, Dirac-source effect, and impact ionization effect. **a** MoS_2_ NC-FET. Reproduced with permission. Reference [211] Copyright 2020, Wiley-Blackwell. **b** CIPS/MoS_2_ vdW NC-FET. Reproduced with permission. Reference [212] Copyright 2019, Springer Nature. **c** MoS_2_ NC-FET. Reproduced with permission. Reference [213] Copyright 2018, Springer Nature. **d** MoS_2_/h-BN/graphene/CIPS vdW FeFET. Reproduced with permission. Reference [32] Copyright 2021, Springer Nature. **e** Atomic threshold switching MoS_2_ FET. Reproduced with permission. Reference [214] Copyright 2021, Wiley–VCH Verlag. **f** MoS_2_ TFET. Reproduced with permission. Reference [215] Copyright 2015, Springer Nature. **g** MoS_2_/Graphene Dirac-source FET. Reproduced with permission. Reference [209] Copyright 2021, American Chemical Society. **h** Nanoscale vertical impact-ionization transistor. Reproduced with permission. Reference [207] Copyright 2020, American Chemical Society. **i** Subthreshold swings of above 2D transistors

SS reduction, capable of enhancing transistor efficiency, is also a focal point in the realm of 2D TMD transistors. Various strategies, each with distinct benefits and drawbacks, have been developed to address this challenge (Fig. 11).

One prevalent strategy involves the creation of NC-FETs [32, 143, 205, 211–213, 216–218] (Fig. 11a–d). These 2D NCFETs incorporate a ferroelectric material in the gate stack, consequently achieving internal voltage amplification and record low SS, breaking the thermal limit of 60 mV decade^−1^. However, the practical implementation of NCFETs with 2D TMDs is still nascent, and acquiring high-quality ferroelectric materials presents difficulties [219]. Further research is required to overcome stability and reliability-related challenges. TFETs offer another viable method for achieving low SS [206, 215, 220] (Fig. 11f). These devices leverage band-to-band tunneling for transistor activation, facilitating an SS lower than the thermal limit at room temperature. However, the fabrication of high-quality tunneling junctions in 2D T-FETs necessitates precise alignment of various 2D materials, complicating the process and limiting practical applications. Impact Ionization FETs (II-FETs) represent another strategy [207, 221] (Fig. 11h). 2D II-FETs employ high-quality 2D heterostructures. Due to the internal gain mechanism in sub-mean-free-path channels, the ballistic impact-ionization process facilitates carrier multiplication, inducing a record low SS during transistor switching. However, fabricating such heterostructures requires precise control over material quality and alignment, limiting potential applications. The novel configuration of Resistive Gate FETs (RG-FETs) has shown promise for achieving extremely low SS [208, 214, 222, 223] (Fig. 11e). 2D RG-FETs combine a memristor and a transistor, with the memristor connected to the transistor's gate electrode. When the threshold voltage is applied to the memristor, it connects the up and down electrodes, transferring the threshold voltage to the transistor's gate. This movement induces a rapid increase in channel carriers, facilitating swift transistor switching and achieving a record low SS. However, integrating a 2D memristor and transistor is complex, thus complicating device fabrication. The memristor connects transistor's drain electrode also can realize low SS transistor [224]. The Dirac-Source FET (DS-FET) configuration provides another measure for SS reduction [209] (Fig. 11g). In this configuration, the source electrode is fabricated from graphene instead of conventional noble metal materials. As graphene possesses a Dirac cone energy band, it induces a sharp change in channel-carrier density during transistor switching, resulting in an SS lower than 60 mV decade^−1^. However, DS-FETs require a precise alignment process, which adds complexity to the device fabrication. Figure 11i shows the quantitative comparation of subthreshold swings. Another way to reduce SS is the Cold-Source FETs, where a “cold” metal is used to replace a conventional metal in a FET contact. Unlike conventional metals, “cold” metals have an energy gap around the Fermi level and function like p- or n-type doped semiconductors. As a result, electrons in this energy region can be effectively filtered out, leading to switching at less than 60 mV decade^−1^ [225–229]. Van der Waals heterojunction field-effect transistors (vdWJFETs) also show promise for SS reduction [230]. However, these FETs involve a complex fabrication process.

In general, we think NC transistors are more compatible and feasible for large-scale production.

Overall, while several strategies exist for reducing SS in 2D TMD transistors, each presents its unique set of challenges. Further research is required to optimize these strategies and develop new approaches that can overcome these limitations, thereby fully realizing the potential of 2D TMDs in power-efficient electronic devices.

### Reduction of Hysteresis Loop

The hysteresis effect is a phenomenon whereby a transistor's output is contingent not only upon its immediate input, but also its historical input. Ideally, output characteristics should trace identical paths during the input voltage's rising and falling phases, thereby eliminating hysteresis. However, practical devices often exhibit hysteresis due to factors such as charge trapping and thermal effects. This effect manifests in the transfer characteristics of transistor operation as a loop when the gate voltage undergoes a bidirectional sweep. The hysteresis loop is integral to defining the stability and reliability of the device. It becomes a critical parameter in applications like memory devices, where the transistor's sustained state is consequential [231].

Although hysteresis can prove beneficial in applications such as memory devices, it is generally undesirable in transistors due to its propensity to induce unpredictable behavior and diminish reliability. In numerous applications, particularly analog and digital circuits, a pronounced hysteresis loop can introduce complications. Hysteresis can engender unpredictable transistor operation, leading to inaccuracies in device operation [232]. Moreover, it can affect the repeatability of transistor characteristics, thereby adversely impacting device reliability and lifespan.

#### Approaches in Hysteresis Reduction in Traditional Semiconductor Transistors

##### Optimization of Gate Dielectric Quality

This approach involves enhancing the quality of the gate dielectric material to diminish the quantity of charge traps. High-quality dielectrics, such as high-k materials, have demonstrated promising outcomes in hysteresis reduction [137]. However, integrating these materials into the device fabrication process while maintaining overall device performance presents challenges. Employment of Passivation Layers: This technique utilizes passivation layers to decrease the density of interface traps [233]. While somewhat effective, these layers can introduce other defects or modify other device parameters. Post-Fabrication Treatments: Techniques such as light or thermal annealing are applied post-fabrication to ameliorate or mitigate the impact of traps [234]. However, these treatments can introduce other defects or modify other device parameters, potentially impairing device performance. Use of Encapsulation Layers: Encapsulation layers serve to protect the active semiconductor layer from environmental factors that may induce hysteresis [235]. However, selecting an appropriate encapsulation material and integrating it into the device structure can be challenging.

Various strategies have demonstrated some success in hysteresis reduction for traditional semiconductor devices, each presents its own set of challenges. Further research is required to optimize these strategies and develop novel ones to effectively reduce hysteresis without compromising other aspects of device performance.

#### Approaches in Hysteresis Reduction in 2D Transistors

The hysteresis loop plays a pivotal role in transistor operation and significantly influences device reliability. While current strategies have achieved some success in reducing hysteresis, it remains a formidable challenge due to potential drawbacks such as additional fabrication complexity or potential degradation of other device parameters. Understanding and controlling the hysteresis effect is crucial for the reliable operation of 2D TMD transistors. Ongoing research efforts are focused on developing strategies to minimize hysteresis and enhance the reliability and predictability of these promising devices.

Transistors based on 2D TMDs are not exempt from hysteresis effects. The distinctive properties of 2D TMDs, such as their atomic thinness and high surface-to-volume ratio, can amplify hysteresis by heightening susceptibility to surface charge trapping. Within the context of 2D TMD-based transistors, the hysteresis loop is primarily attributed to charge trapping at the interface of the 2D material and the dielectric, or within the dielectric itself [236]. These trapped charges can alter the device's threshold voltage, engendering a shift in the current–voltage characteristics during a voltage sweep and resulting in the observed hysteresis. The presence of hysteresis in 2D TMD transistors can significantly affect device reliability, leading to unstable device operation, especially in digital circuits and analog amplifiers where a predictable and steady response is vital for accurate functioning [60]. To mitigate the hysteresis effect in 2D TMD transistors, several strategies can be employed, including surface passivation [237], dielectric engineering [30], encapsulation [33], and post-fabrication treatments [39].

**Fig. 12:**
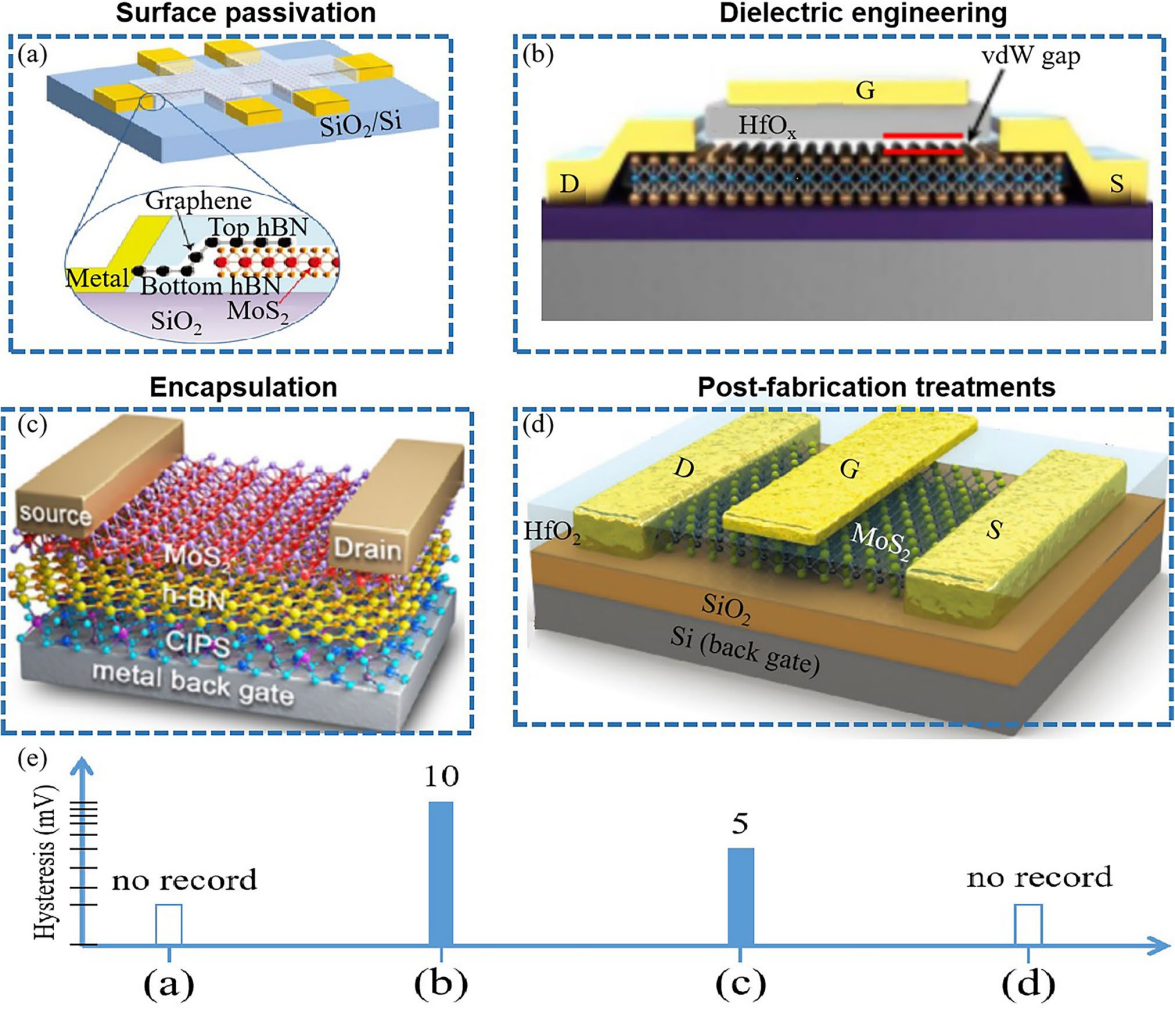
Hysteresis reduction of 2D transistors includes four strategies such as surface passivation, dielectric engineering, encapsulation, and post-fabrication treatments. **a** MoS_2_ FET with hBN passivation layer. Reproduced with permission. Reference [238] Copyright 2015, Springer Nature. **b** MoS_2_ FET with HfO_x_ dielectric. Reproduced with permission. Reference [239] Copyright 2022, Springer Nature. **c** MoS_2_ negative-capacitance FET with h-BN/CuInP_2_S_6_ dielectric. Reproduced with permission. Reference [33] Copyright 2023, American Chemical Society. **d** Single-layer MoS_2_ FET with post-fabrication treatment. Reproduced with permission. Reference [39] Copyright 2011, Springer Nature. **e** Hysteresis of above 2D transistors

Several methodologies have been devised and implemented to diminish hysteresis in traditional transistor operation, each presenting varying degrees of efficacy and associated challenges. Addressing hysteresis in 2D TMD transistors is crucial for enhancing device performance and reliability. Several techniques have been explored to mitigate hysteresis (Fig. 12), each with varying degrees of efficacy and associated challenges:

##### Surface Passivation

This technique involves applying a passivating layer to shield the 2D TMD material from environmental factors such as moisture and oxygen. This can significantly reduce hysteresis by minimizing the number of trap states that interact with the charge carriers [237, 238] (Fig. 12a). However, selecting an appropriate passivating material and creating a uniform layer without introducing additional defects can be challenging. Dielectric engineering: The choice of dielectric material can significantly influence hysteresis. High-k dielectrics can reduce hysteresis by minimizing the trapping and de-trapping of charge carriers at the interface [30]. However, integrating these materials can increase the complexity of the fabrication process and potentially introduce additional defects. A recent study highlighted the contribution of the exchange of electrons between oxide and channel layers to hysteresis. Thus, understanding and controlling this exchange can facilitate hysteresis reduction. In this regard, van der Waals-gap-gated transistors with an additional air-gap between dielectric and channel layers have been demonstrated [240], exhibiting negligible hysteresis of 10 mV [239] (Fig. 12b). However, the fabrication process is complex. Encapsulation: Encapsulation with an inert material such as h-BN can protect the 2D TMD from environmental factors and reduce charge trapping at the interface, thereby reducing hysteresis [33, 241] (Fig. 12c). However, the fabrication of high-quality encapsulation layers can be challenging. Post-fabrication treatments: Treatments such as annealing, applied post-fabrication, can reduce hysteresis by removing adsorbed species and reducing the number of trap states [39] (Fig. 12d). However, such treatments can also cause damage to the 2D TMD material, potentially degrading device performance. Figure 12e shows the quantitative comparation of hysteresis.

In general, we think encapsulation technique is more compatible and feasible for large-scale production.

Several techniques can be used to reduce hysteresis in 2D TMD transistors, each presents its own set of challenges. Further research is required to optimize these techniques and develop novel methods that effectively mitigate hysteresis while maintaining device performance and reliability.

### Enhancement of Carrier Mobility

Carrier mobility, referring to the velocity at which charge carriers (either electrons or holes) traverse a semiconductor material under an electric field's influence, is crucial for the efficient operation of transistors [242]. It directly impacts the transistor's on-current, and by extension, the device speed, influencing the clock speed of microprocessors where transistors serve as switches [243]. Consequently, augmenting carrier mobility is a fundamental strategy in amplifying electronic device speed [244]. Additionally, carrier mobility exerts influence on a device's power efficiency. Higher mobility transistors necessitate lesser current for state transitions, leading to reduced power consumption [245]. This aspect is particularly crucial in portable electronic devices where energy efficiency and extended battery life are paramount.

Numerous factors, including the intrinsic properties of the semiconductor material, the quality of material interfaces, and the operating temperature, govern carrier mobility. Advancements in material synthesis and device fabrication techniques have facilitated significant enhancements in carrier mobility. In the realm of transistors based on 2D TMDs, carrier mobility remains a critical determinant of device performance. Elevated carrier mobility permits swifter transitions between "on" and "off" states, enabling high-speed operation of the transistor. This functionality is especially essential in high-frequency applications, such as radio frequency (RF) circuits and high-speed digital circuits [151]. Various factors influence carrier mobility in 2D TMDs, including the material quality, the presence of defects and impurities, and the interaction between the 2D material and the surrounding environment [246]. High-quality, defect-free 2D TMDs exhibit superior carrier mobility, contributing to improved transistor performance. Interestingly, 2D TMDs can display a range of carrier mobilities contingent on the specific material and its thickness. For example, monolayer MoS_2_, a frequently studied 2D TMD, reportedly has carrier mobilities ranging from 10 to 100 cm^2^ v^−1^ s^−1^, whereas bulk MoS_2_ has higher carrier mobility around 200 cm^2^ v^−1^ s^−1^ [39, 247]. Achieving high carrier mobility can be challenging due to various material and fabrication factors. However, much research is focused on strategies to enhance carrier mobility in 2D TMD transistors, including materials selection [248], improving material quality [60], surface functionalization [249], dielectric engineering [131], strain engineering [34], and device architecture optimization [35].

Carrier mobility is an essential factor for efficient and high-speed transistor operation. Continued exploration of new materials and device structure optimization is required to further enhance carrier mobility, aiming to improve the speed and power efficiency of electronic devices.

#### Recent Advancement in Carrier Mobility Enhancement in Traditional Semiconductor Transistors

Recent years have seen significant advancements in enhancing carrier mobility in conventional semiconductor devices. These advancements owe much to improvements in materials science, device engineering, and fabrication techniques. New device architectures, such as FinFETs and nanowire transistors, have been engineered to augment carrier mobility by improving electrostatic control and mitigating short-channel effects [250, 251]. However, these advancements present challenges in the form of complex fabrication processes and the need for meticulous alignment and control. The quality of the semiconductor-dielectric interface profoundly influences carrier mobility through trap-induced scattering and trapping. Techniques like surface passivation, the use of high-k dielectrics, and atomic layer deposition have been employed to improve interface quality [252]. These methods, however, may introduce other defects or alter other device parameters. The application of strain to semiconductors can modulate their band structure, resulting in enhanced carrier mobility. This method has been widely used in silicon-based devices, but the precise control of strain remains challenging [253]. High temperatures can impair carrier mobility. Advances in thermal management, such as the usage of thermal interface materials and advanced cooling techniques, have contributed to maintaining high mobility [254]. Yet, integrating these solutions into miniaturized devices poses a significant challenge.

Enhancing carrier mobility is a complex issue that necessitates advances in materials, device design, and fabrication techniques. Despite recent progress, considerable challenges persist. Continued interdisciplinary research is required to overcome these obstacles and realize the full potential of high-mobility transistor devices.

#### Recent Advancement in Carrier Mobility Enhancement in 2D Transistors

**Fig. 13:**
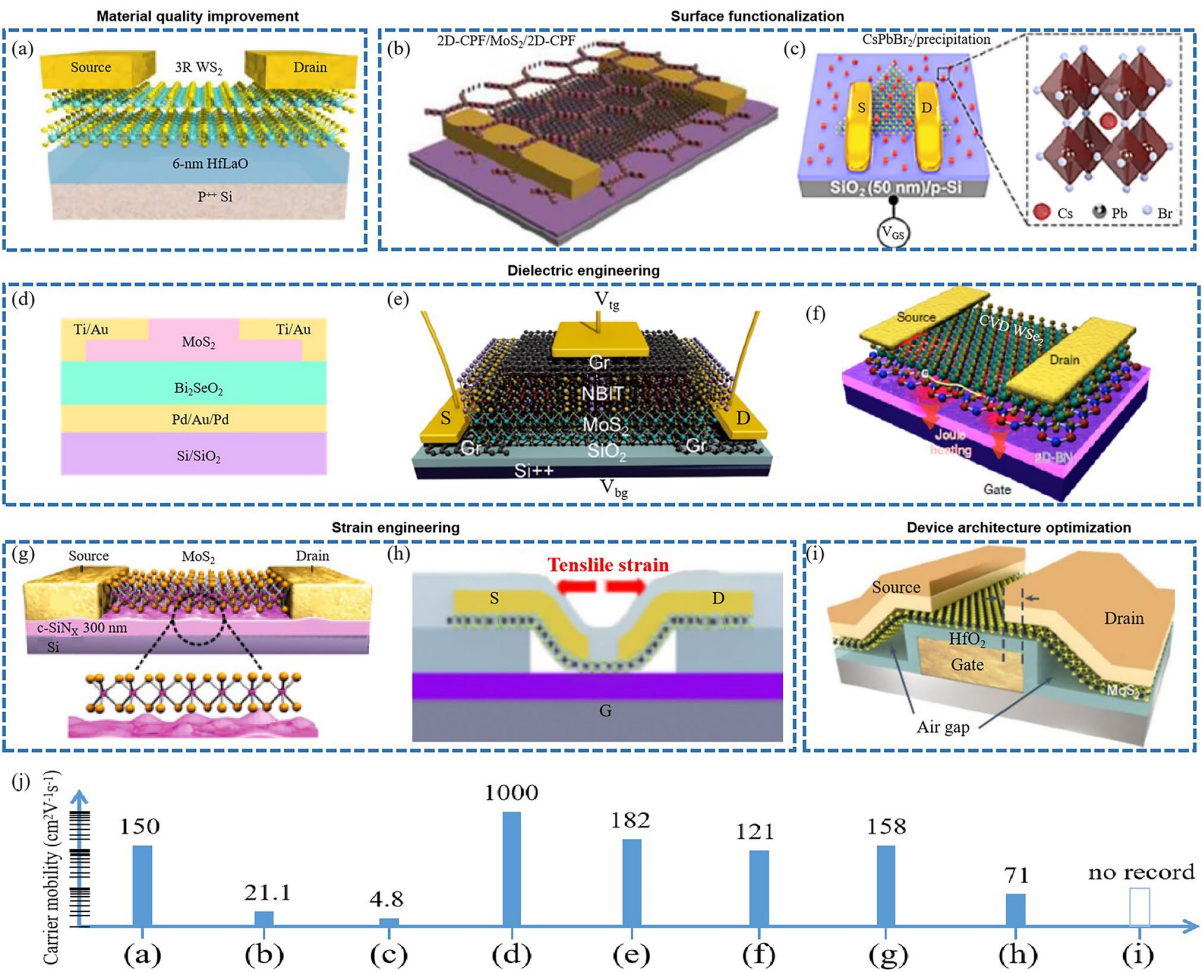
Carrier mobility enhancement in 2D transistors involves material quality improvement, surface functionalization, dielectric engineering, strain engineering, and device architecture optimization. **a** Rhombohedral-stacked bilayer WS_2_ FET. Reproduced with permission. Reference [255] Copyright 2023, American Association for the Advancement of Science. **b** MoS_2_ FET coupled with 2D organic frameworks. Reproduced with permission. Reference [256] Copyright 2023, Wiley-Blackwell. **c** CsPbBr_3_ precipitation on a MoS_2_ FET. Reproduced with permission. Reference [249] Copyright 2023, American Chemical Society. **d** MoS_2_ FET with high dielectric constant of Bi_2_SeO_2_. Reproduced with permission. Reference [131] Copyright 2023, Springer Nature. **e** Dual-gate MoS_2_ FeFET. Reproduced with permission. Reference [257] Copyright 2020, Wiley-Blackwell. **f** WSe_2_ FET using conformal BN dielectric interface. Reproduced with permission. Reference [150] Copyright 2019, Springer Nature. **g** Crested two-dimensional transistors. Reproduced with permission. Reference [258] Copyright 2019, Springer Nature. **h** Strained MoS_2_ transistor. Reproduced with permission. Reference [34] Copyright 2023, American Chemical Society. **i** MoS_2_ transistor with air-gap structure. Reproduced with permission. Reference [151] Copyright 2023, Springer Nature. **j** Carrier mobilities of above 2D transistors

The enhancement of carrier mobility in 2D-TMD transistors has been the focus of numerous recent research efforts, given its crucial role in determining device performance. Various advancements and methodologies have been proposed (Fig. 13), each presenting unique challenges.

##### Material Quality Improvement

Enhancing the quality of 2D TMD materials is a direct approach to augment carrier mobility. High-quality, defect-free materials reduce scattering of charge carriers, leading to improved mobility [259]. Growth techniques, such as chemical vapor deposition (CVD), have been optimized to yield high-quality 2D TMDs [60]. Moreover, the growth of rhombohedral-stacked bilayer transition metal dichalcogenides can significantly improve mobility [255] (Fig. 13a). However, achieving large-scale, uniform, and defect-free growth remains a challenge. Surface functionalization: Surface functionalization can modulate the properties of 2D TMDs. For instance, molecular doping with organic molecules or metal chalcogenides can introduce additional charge carriers and thereby enhance mobility [249, 256, 260, 261] (Fig. 13b, c). While promising, the stability and reproducibility of these functionalization techniques warrant further investigation. Dielectric engineering: The choice of dielectric material can significantly impact carrier mobility. High-k dielectrics can lead to reduced impurity scattering and improved mobility [131, 143, 216–218, 257, 262–264] (Fig. 13d, e). Additionally, a conformal hexagonal-boron nitride dielectric layer with an atomically clean interface can enhance the mobility of 2D transistors [150] (Fig. 13f). However, integrating these materials without introducing interface traps is challenging. Strain engineering: Strain engineering is a novel approach for mobility enhancement. By applying mechanical strain, the band structure of 2D TMDs can be modified and electron–phonon coupling can be suppressed, leading to increased carrier mobility [34, 258, 265, 266] (Fig. 13g, h). The challenge lies in precisely controlling the strain to avoid material degradation. Device architecture optimization: The design of the transistor itself can play a role in enhancing carrier mobility. For example, 2D fin field-effect transistors allow for better control over the charge carrier concentration, leading to improved mobility [35]. Furthermore, MoS_2_ transistors with an air-gap structure exhibit low resistance due to doping-free ohmic contacts and low parasitic capacitance due to the low dielectric permittivity in the air-gap region. This results in enhanced mobility and contributes to high operation speed [151] (Fig. 13i). However, fabricating such complex structures requires advanced fabrication techniques. Figure 13j shows the quantitative comparation of carrier mobilities.

In general, we think dielectric-engineering technique is more compatible and feasible for large-scale production.

Substantial advancements have been made in enhancing carrier mobility in 2D TMD transistors, each approach presents its unique set of challenges. Additionally, these kinds of devices may involve some alleged issues in determining high mobilities [242]. Further research is necessary to overcome these challenges and establish the groundwork for high-performance electronic devices utilizing 2D TMDs.

### Enhancement of On/Off Ratio

The on/off ratio of a transistor, defined as the ratio of the current that passes through the device in its "on" state to that in its "off" state, is a crucial determinant of transistor performance and energy efficiency. A high on/off ratio, which signifies a robust distinction between the "on" and "off" states, is desirable for a multitude of reasons.

Primarily, a high on/off ratio is critical for binary logic operations executed by digital circuits. It ensures the transistor's effective state-switching capability, thereby minimizing signal errors and bolstering overall circuit performance.

Secondly, within a transistor, a high on/off ratio facilitates precise control of electron flow, mitigating energy wastage and enhancing switching applications' efficiency. Ideally, no current should flow when a transistor is in the "off" state. However, due to factors such as device imperfections and quantum mechanical tunneling, a small leakage current invariably exists. A high on/off ratio ensures this leakage is minimized, leading to enhanced energy efficiency [267]. This is particularly significant for battery-powered devices where power consumption minimization is paramount.

Improvements in the on/off ratio can be attained through several methods. Material selection is pivotal. Semiconductors with a larger bandgap typically exhibit superior on/off ratios due to decreased off-state leakage [268]. Likewise, device architecture significantly influences this ratio. Scaled-down devices like FinFETs and nanowire transistors exhibit higher on/off ratios by enhancing electrostatic control [250]. Additionally, threshold voltage tuning, accomplished through doping concentrations or gate dielectric materials, can optimize the on/off ratio [123]. Despite these advancements, challenges persist. Maintaining a high on/off ratio while simultaneously achieving other desirable device characteristics such as high carrier mobility, low power consumption, and small device dimensions, poses a consistent challenge in transistor design.

The enhancement of the on/off ratio in 2D TMD transistors is an active research area. Techniques like gate engineering [28, 35], contact optimization [80], defect control [269, 270], and device architecture optimization [35] have been employed to improve the on/off ratio, thereby enhancing transistor performance and energy efficiency. However, trade-offs exist. For instance, an increase in the on/off ratio may cause a reduction in carrier mobility, potentially impairing the transistor's switching speed [1]. Striking a balance between these parameters is critical for optimizing transistor design.

The on/off ratio is a critical parameter in determining the performance and energy efficiency of 2D TMD-based transistors. Various strategies, such as bandgap adjustment, carrier concentration modulation, and insulating layer capacitance variation, can optimize the on/off ratio and enhance transistor performance. Nonetheless, optimizing this ratio without compromising other device characteristics remains a challenge. Continued research and innovation in materials, device design, and fabrication techniques are necessary to address these challenges.

#### Strategies to Enhance the On/Off Ratio of Traditional Semiconductor Transistors

Enhancing the on/off ratio in traditional transistors is pivotal for improving energy efficiency and overall performance. Various strategies have been implemented to this end, each presenting its own success rate and inherent challenges.

##### Material Selection

Semiconductors possessing larger bandgaps, such as gallium nitride (GaN) and silicon carbide (SiC), generally exhibit higher on/off ratios due to a decrease in off-state leakage [271]. However, the application of these materials introduces challenges, including elevated material and manufacturing costs, as well as issues related to material purity and crystal defects [272].

##### Device Architecture

The adoption of advanced device architectures, including Fin Field-Effect Transistors (FinFETs) and nanowire transistors, can enhance electrostatic control, thereby increasing the on/off ratio [250]. Nevertheless, these architectures introduce fabrication challenges, such as amplified process complexity and potential for short-channel effects as devices are scaled down [56].

Threshold voltage tuning: Modifying a transistor's threshold voltage, through strategies such as varying doping concentrations or altering the gate dielectric material, can increase the on/off ratio [56]. However, this approach may render the device more susceptible to variations in manufacturing processes, thereby affecting device uniformity. Alternative Transistor Designs: Designs such as TFETs and NCFETs have been explored for their potential to yield high on/off ratios [79, 202]. However, these designs face challenges, such as achieving steep subthreshold slopes and controlling ferroelectric material properties, respectively.

While numerous strategies for enhancing the on/off ratio exist, each introduces its own set of challenges. Continued research and technological advancements are necessary to overcome these obstacles and further improve transistor performance.

#### Strategies to Enhance the On/Off Ratio of 2D Transistors

The on/off ratio is a crucial parameter for evaluating transistor performance. Higher values facilitate clearer signal distinction and improved energy efficiency. The following sections discuss the strategies employed to enhance the on/off ratio in transistors based on 2D TMDs, their effectiveness, and associated challenges.

**Fig. 14:**
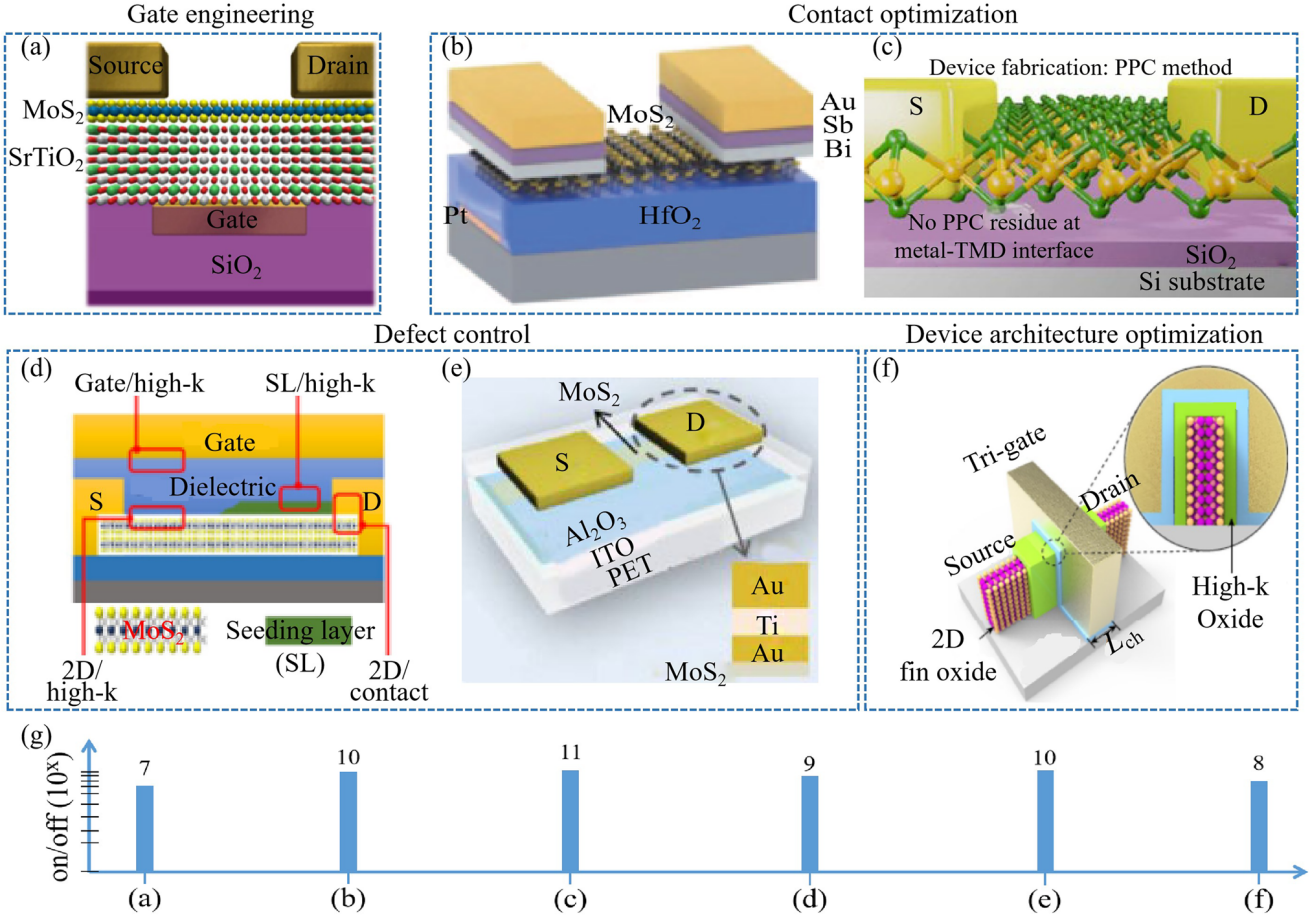
Strategies to enhance the on/off ratio of 2D transistors involve gate engineering, contact optimization, defect control, device architecture optimization. **a** High-κ perovskite membranes as insulators for two-dimensional transistors. Reproduced with permission. Reference [30] Copyright 2022, Springer Nature. **b** Bi-contacted MoS_2_ field effect transistors. Reproduced with permission. Reference [273] Copyright 2024, Science China Press. **c** High performance MoS_2_ transistor fabricated via residue-free transfer. Reproduced with permission. Reference [191] Copyright 2023, Springer Nature. **d** Defect control for 2D transistors. Reproduced with permission. Reference [23] Copyright 2021, Springer Nature. **e** High performance MoS_2_ transistor fabricated via modified chemical vapor deposition. Reproduced with permission. Reference [274] Copyright 2020, Springer Nature. **f** 2D fin field-effect transistors. Reproduced with permission. Reference [35] Copyright 2023, Springer Nature. **g** The on/off ratios of above 2D transistors

The main strategies to enhance the on/off ratio of 2D transistors including gate engineering, contact optimization [195], defect control, and device architecture optimization (Fig. 14). Gate engineering is a widely used strategy for enhancing the on/off ratio. By optimally designing the gate dielectric and its interface with the channel, the gate's control over the channel can be improved, resulting in a higher on/off ratio [28, 30, 145, 264] (Fig. 14a). However, this approach necessitates precise control over material deposition and interface properties, which may be challenging to consistently achieve. Enhancing the contact between the metal electrodes and the 2D TMDs can significantly improve the on/off ratio [166, 273] (Fig. 14b). Employing metals with suitable work functions can reduce contact resistance and enhance device performance [166]. Clean contact between the metal electrodes and the 2D TMDs also can increase the on/off ratio. However, fabricating high-quality contacts on 2D materials is a complex task, often involving intricate deposition and annealing processes. Recently, the PPC-assisted transfer method demonstrates its promising for high-quality contacts. As shown in Fig. 14c, the PPC-assisted transfer method has been employed for high on/off MoS_2_ transistor due to the clean contact, benefiting from its residue-free transfer effect [191] (Fig. 14c). Defects in 2D TMDs can trap charge carriers and degrade device performance. By controlling the growth process to minimize defects, a higher on/off ratio can be achieved [23] (Fig. 14d). However, obtaining large-area, defect-free 2D TMDs remains a significant challenge. Recently, the modified chemical vapour deposition process has shown its prospect for large-area, defect-free 2D TMDs. As depicted in Fig. 14e, the modified chemical vapour deposition process has been used for high on/off MoS_2_ transistor [274] (Fig. 14e). The on/off ratio can also be enhanced by optimizing the device architecture. For instance, 2D fin-field-effect-transistor structures provide superior control over the channel, resulting in improved on/off ratios [35] (Fig. 14f). However, fabricating such structures can be complex and necessitates advanced lithography techniques. Figure 14g shows the quantitative comparation of on/off ratios.

In general, we think contact-optimization technique is more compatible and feasible for large-scale production.

Various strategies have been employed to enhance the on/off ratio in 2D TMD transistors, each brings its own set of challenges. Further research is needed to refine these techniques and develop new strategies for device optimization.

So far, we have introduced the key parameters in 2D TMD transistors and their optimization methods. For intuition, comparison of performance limits in 2D TMD transistors has been demonstrated (Table 1) [27, 28, 30, 31, 33–35, 66, 93, 130, 156, 171, 191, 212, 214, 239, 249, 274].

**Table 1:** Comparison of key parameters in 2D TMD transistors

Channel layer	Channel length (nm)	Gate length (nm)	Contact length (nm)	Dielectric thickness (nm)	Contact resistance	Subthreshold swing (mV dec^−1^)	Hysteresis loop (mV)	Carrier mobility (cm^2^ V^−1^ s^−1^)	On/Off ratio	References
MoS_2_	0.65	~	~	300	~	~	~	~	10^3^	[27]
MoS_2_	2	~	~	300	~	~	~	~	5 × 10^5^	[68]
MoTe_2_	~	< 2	~	300	~	~	~	~ 30	~ 10^5^	[93]
MoS_2_	~	0.34	~	14	~	117	~	~	~ 10^5^	[28]
MoS_2_	~	~	< 2	~	10^−6^ Ω cm^−2^	~	~	~	~ 10^6^	[29]
MoS_2_	~	~	< 1	~	~	116	~	~ 30	~ 10^6^	[113]
MoS_2_	~	~	~	0.67	~	~	~	~	> 10^6^	[130]
MoS_2_	~	~	~	< 1	~	70	~	~	> 10^7^	[30]
MoS_2_	20	~	~	~	42 Ω μm^−1^	~	~	50	> 10^6^	[31]
InSe	10	~	~	2.6	62 Ω μm^−1^	75	~	~	> 10^7^	[171]
MoS_2_	~	~	~	~	~	3.9	~	~	~ 10^7^	[215]
MoS_2_	~	~	~	~	~	4.97	~	~	~ 10^6^	[211]
MoS_2_	~	~	~	~	~	62	5	~	~ 10^7^	[33]
MoS_2_	~	~	~	~	~	63.1	10	~	~ 10^7^	[239]
MoS_2_	~	~	~	~	~	~	~	> 1000	~ 10^9^	[131]
MoS_2_	~	~	~	~	~	~	~	182	~ 10^6^	[257]
MoS_2_	~	~	~	~	78 Ω μm^−1^	~	~	19.2	~ 10^11^	[191]
MoS_2_	~	~	~	~	2.9 kΩ μm^−1^	~	~	55	~ 10^10^	[274]

## Realization of P-Type Transistors, Single Logic Transistor and Memory Devices

By pushing the dimension and performance limits of 2D TMDs based transistors, the single 2D TMD transistor reaches an optimal state. After that, the single 2D TMD transistors show many potential applications, which includes three main directions: P-type transistors, single logic transistor and memory devices.

### Strategies to Realize P-Type Transistors

2D TMDs, including MoS_2_, WS_2_, WSe_2_, and MoTe_2_, exhibit layer-dependent electronic properties. Their high on/off ratios and low subthreshold swings position them as compelling alternatives to conventional semiconductors for the fabrication of transistors with atomic-scale thickness [1]. Advancing CMOS technology, the backbone of modern digital circuits, covers pairs of n- and p-type FETs [159, 276]. However, 2D TMDs can be used for fabricating n-type transistors easily, and hard to fabricate p-type 2D TMD transistor. Hence, p-type transistors have consequently been the focus of extensive research.

**Fig. 15:**
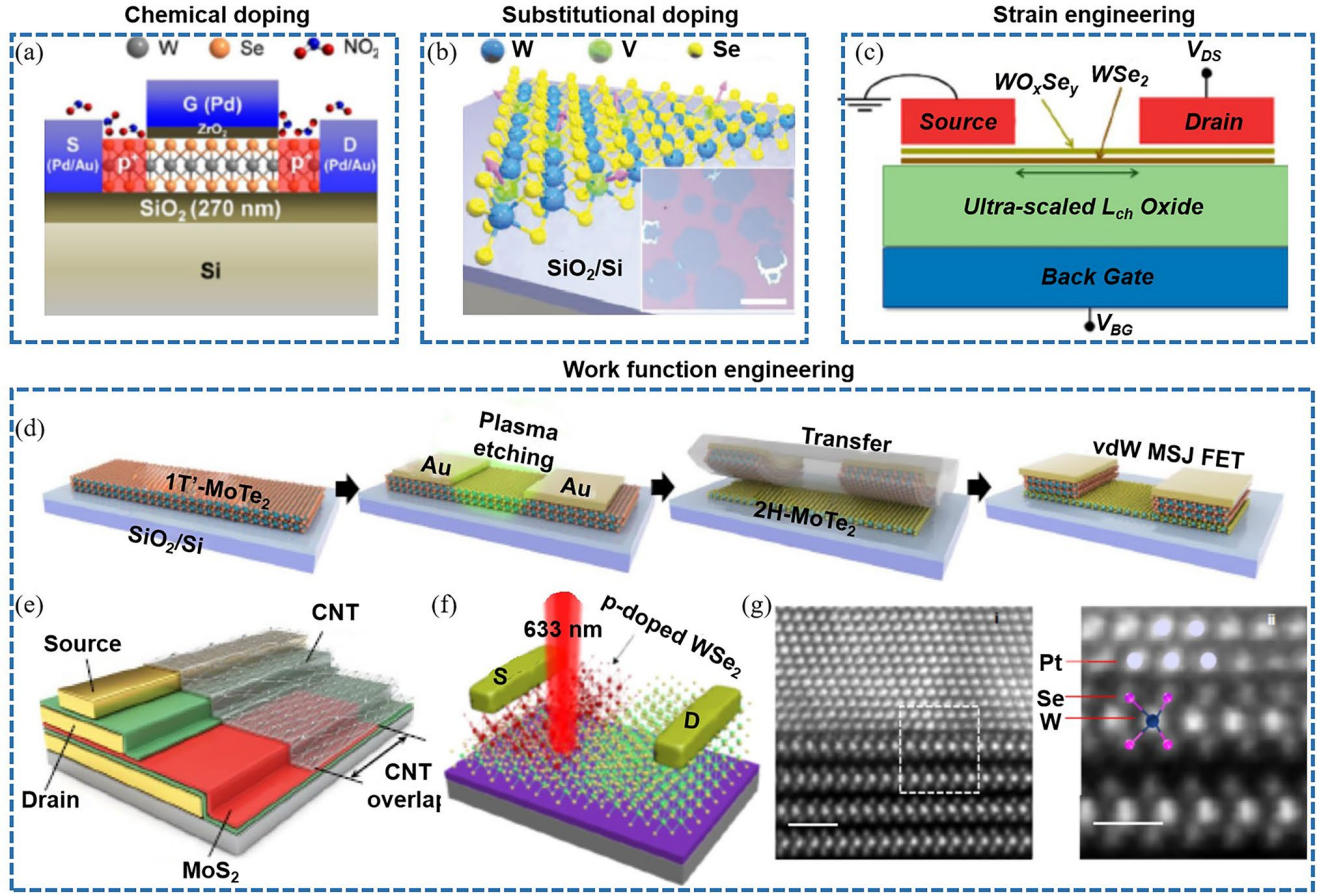
P-type transistors. **a** NO_2_ molecules for p-type dopants of WSe_2_. Reproduced with permission. Reference [36] Copyright 2022, Wiley–VCH Verlag. **b** V-doped WSe_2_ by mixing W with V precursors. Reproduced with permission. Reference [290] Copyright 2020, Wiley–VCH Verlag. **c** Ultrascaled p-type FET based on WSe_2_/WO_x_Se_y_ heterostructure. Reproduced with permission. Reference [291] Copyright 2023, American Chemical Society. **d** The p-type MoTe_2_-based transistor fabrication. Reproduced with permission. Reference [176] Copyright 2023, Springer Nature. **e** The MoS_2_/CNT heterojunction transistor. Reproduced with permission. Reference [292] Copyright 2023, Springer Nature. **f** WSe_2_ FET after laser scanning. Reproduced with permission. Reference [285] Copyright 2019, American Chemical Society. **g** Atomic resolution images of Pt on multilayer WSe_2_. Reproduced with permission. Reference [159] Copyright 2022, Springer Nature

Recent research has demonstrated promising outcomes through the deployment of various techniques, such as chemical doping [36], substitutional doping [277], strain engineering [278], work function engineering [105], and reconfigurable transistors [275, 279–284] (Fig. 15), each presenting its own advantages and associated difficulties. Chemical doping involves introducing a p-type dopant into the TMD material to modify its electrostatic properties [36, 285–289] (Fig. 15a). Despite its utility, this approach may disrupt the lattice structure of 2D TMDs, potentially affecting electrical properties. Furthermore, ensuring accuracy and control over the doping concentration poses challenges. The substitutional doping technique entails the replacement of specific atoms in the TMD material with p-type elements [277]. Successful p-type WSe_2_ FETs have been achieved via V substitutional doping [290] (Fig. 15b). While this method preserves the lattice structure, the doping process can be intricate and demands precise control to ensure uniform substitution. As a more recent approach, strain engineering applies mechanical strain to the TMD material to alter its band structure, potentially inducing a transition from n-type to p-type behavior [278, 291] (Fig. 15c). However, the control and maintenance of the applied strain are challenging, and the strain may induce defects in the TMD material. Work function engineering is another promising strategy that aims to realize p-type TMDs by modifying the work function of the metal contacts to align with the valence band of TMDs, thereby facilitating hole injection [105, 154, 159, 176, 292, 293] (Fig. 15d–g). However, identifying suitable metals that can withstand the fabrication process and deliver stable performance remains challenging. Reconfigurable transistors with polarity gate and control gate have the capability to form p-type transistor. However, their fabrication process is complexity than conventional transistors.

In general, we think 2D reconfigurable transistors are more compatible and feasible for large-scale production.

Various strategies for realizing p-type transistors in 2D TMDs have been explored, each strategy presents its own set of challenges. Further research is required to refine these techniques and to develop new methodologies [294, 295] for successful implementation of p-type transistors in 2D TMDs.

### Strategies to Realize Single Logic Transistor

The dawn of single logic transistors, particularly those based on 2D TMDs, has revolutionized the landscape of electronics, fostering a new era of miniaturized, high-performance, and energy-efficient devices. These atomically thin semiconductors are challenging traditional norms of electronic computations and logic gate designs, suggesting the potential for executing more complex logic operations with fewer transistors. Historically, silicon-based CMOS technology has been the cornerstone of digital electronics, necessitating multiple transistors to construct even the simplest logic gates [296]. For example, traditional silicon transistors, which can only be regulated from the top surface, require two top-gated transistors to implement a two-input logic gate. However, the emergence of single logic transistors utilizing 2D TMDs presents the potential for a significant reduction in component count [87].

**Fig. 16:**
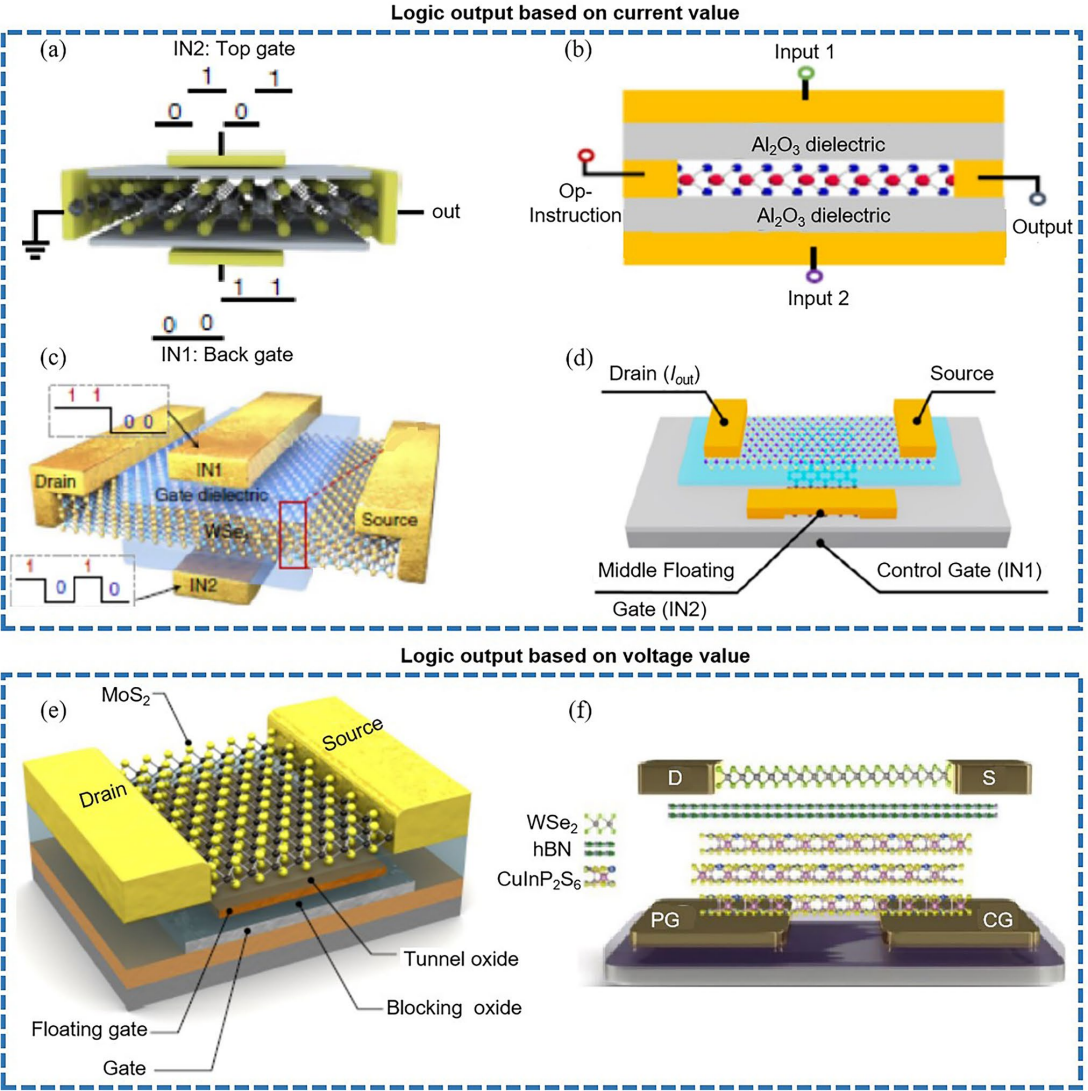
Single logic transistor. **a** Dual-gate MoS_2_ transistor. Reproduced with permission. Reference [126] Copyright 2019, Springer Nature. **b** Dual-gate WSe_2_ transistor. Reproduced with permission. Reference [297] Copyright 2022, Springer Nature. **c** Dual-gate WSe_2_ transistor. Reproduced with permission. Reference [37] Copyright 2021, Springer Nature. **d** WSe_2_ floating gate transistor. Reproduced with permission. Reference [298] Copyright 2023, American Chemical Society. **e** MoS_2_ floating gate transistor. Reproduced with permission. Reference [299] Copyright 2020, Springer Nature. **f** Reconfigurable van der Waals ferroelectric transistor. Reproduced with permission. Reference [281] Copyright 2023, American Chemical Society

The development of single logic transistors based on 2D TMDs represents a transformative shift in the field of electronics. Achieving single logic transistors has been a major focus in recent years, with several strategies being employed to enhance their effectiveness (Fig. 16). It is important to note that the logic results of a single logic transistor can be determined in two ways: current and voltage.

The employment of 2D TMDs in these transistors offers additional benefits. TMDs are renowned for their superior electronic properties, including high carrier mobility and on/off ratio, as well as their mechanical flexibility and robustness. These attributes render TMD-based transistors especially suitable for next-generation electronics, including flexible and wearable devices. Recently, promising strategies for achieving high-performance single 2D logic transistors have been proposed, such as dual-gate transistors [37, 126, 297, 300] (Fig. 16a–c), FGFET [298] (Fig. 16d–f), vdW FeFETs [301], and ferroelectric split-gate transistors [281]. Despite the intriguing potential, the journey to fully exploit the capabilities of single 2D logic transistors is laden with challenges. These include maintaining material quality, managing contact resistance, and integrating with existing technologies. Furthermore, the fabrication of TMD-based devices often involves complex procedures that need optimization for large-scale production.

Nonetheless, the pursuit of single logic transistors based on 2D TMDs continues to inspire innovative techniques for implementing complex logic operations with fewer transistors. Their unique properties and capabilities hold the potential to drive significant breakthroughs in various fields, including computing, imaging, and wearable technology, heralding an exciting future for nanoelectronics.

#### Logic Output Based on Current Value

One promising approach involves the development of a MoS_2_ dual-gate transistor, serving as a single logic transistor where the top and bottom gates function as two input terminals. This transistor realizes OR and AND logic functions, simplifying device structure and enhancing area efficiency. Additionally, the single logic transistor can be modulated by photo illumination [126]. Another strategy demonstrates a single WSe_2_ double-gated transistor achieving AND and XNOR logic functions. This transistor can serve as a pixel processing unit in an image processing array, performing various image tasks while using less than 16% of the transistors required by traditional circuits [297]. Huawei Chen et al. have fabricated various single 2D double-gated transistors, including WSe_2_, BP, and MoS_2_ double-gated transistors, capable of executing XNOR, NOR, and AND gates, respectively. Notably, a logic half-adder based on these XNOR and AND gates can save 78% of the area compared to traditional circuit design [37]. Weihui Sang et al. have explored dual-gate Gaussian-type transistors based on a MoS_2_/BP heterojunction. This single logic device with electrically driven reconfigurability can realize all fundamental Boolean logic operators, resulting in a transistor consumption that is only 13% of traditional circuit designs [300]. Xinzhu Gao et al. have prepared a 2D ferroelectric field-effect transistor (Fe-FET) based on the CuInP_2_S_6_/WS_2_ van der Waals heterostructure. This 2D Fe-FET can be used as a single logic transistor to realize the AND logical operation [302]. Zhe Sheng et al. have fabricated a floating gate field-effect transistor (FGFET) based on a WSe_2_/BN/Graphene heterostructure. The FGFET serves as a single logic transistor, achieving AND/XNOR lo gic circuits with a significant reduction in transistor numbers compared to traditional devices [298]. Jingjie Niu et al. have fabricated van der Waals ferroelectric field-effect transistors (vdW FeFETs) based on SnS/BN/CuInP_2_S_6_ heterostructures. The single vdW FeFET can operate AND, NAND, XOR logical functions [301]. Ruixuan Peng et al. have explored a single-gate MoTe_2_ reconfigurable logic transistor with programmable graded doping, achieving the NOR logic function [303]. Ruge Quhe et al. have fabricated the AND, OR logical gates based on a double-gated α-In_2_Se_3_ transistor [304]. Tao Zhu et al. have constructed the WSe_2_/Ta_2_NiSe_5_ heterostructure logic devices with input terminals including light on/off, polarization angles bias voltage, and gate voltage [305].

#### Logic Output Based on Voltage Value

Ankita Ram et al. have prepared a reconfigurable ferroelectric split-gate transistor based on WSe_2_/BN/CuInP_2_S_6_, capable of realizing XNOR, AND, and NAND logic circuits [281]. Guilherme Migliato Marega et al. have developed MoS_2_-based FGFETs capable of realizing inverter logic [299]. Another study revealed that a ternary inverter logic function can be realized using a single 2D van der Waals vertical heterojunction transistor and a resistor [306]. It's important to note that voltage logic output can be derived from a single transistor and a resistor, whereas current logic output can only be derived from the single transistor. Recently, Yu et al. have fabricated J-MISFET by vertically stacking a junction transistor onto a metal–insulator–semiconductor (MIS) transistor. The J-MISFET can be used for constructing high-gain inverter, NAND, and NOR logic gates [307].

However, these strategies come with their own set of challenges. Maintaining the quality of 2D materials during device fabrication is crucial, as defects in the atomic layers can negatively impact the transistor's performance [103]. Contact resistance between the transistor and the metal electrodes impacts device performance [31, 173]. The monotonic increase in the drain current of MoS_2_-based transistors has limited the reconfigurable logic gate operations [39]. Variability in device performance, particularly at the nanoscale, poses a significant obstacle for large-scale device integration and manufacturing [87]. All strategies involve the use of van der Waals heterostructures, requiring multi-step precision alignment, increasing fabrication complexity. Despite these challenges, the potential rewards of successfully developing single 2D logic transistors are significant, heralding a revolution in the field of nanoelectronics. However, overcoming these technical challenges requires continued research, innovation, and refinement of strategies.

In general, we think voltage-output based single logic transistors are more compatible and feasible for large-scale production.

### Performance of 2D TMDs-Based Transistors as Memory Devices

2D monolayer TMDs have drawn considerable interest in electronics due to their distinct attributes, such as direct bandgap, high carrier mobility, and atomic-scale thickness [8, 60]. Notably, employing these materials in memory devices offers several advantages and introduces challenges that differ from their usage in other types of devices. 2D monolayer TMDs offer potential for high-density memory devices due to their atomic thickness, enabling significant miniaturization [98]. Their direct bandgap contributes to low-power operation, a critical factor in memory applications where energy efficiency is paramount [1]. Another benefit is their high on/off ratio, advantageous for memory devices that require a distinct differentiation between logic states [308]. Moreover, 2D TMDs have demonstrated potential in non-volatile memory applications, showcasing excellent endurance, retention, and multi-level cell (MLC) capabilities [38]. Furthermore, the unique material characteristics of 2D TMDs can augment memory device functionality, offering ultrafast memory times [309–311].

Despite these benefits, several obstacles persist in the utilization of 2D TMDs for memory devices. The synthesis of high-quality 2D TMD layers with controlled thickness and minimal defects remains a challenge [18]. Additionally, the integration of these materials into existing semiconductor manufacturing processes often necessitates high temperatures that may degrade the 2D materials [312]. Moreover, issues such as contact resistance and performance variability, which can impact the reliable operation of memory devices, pose significant challenges in the application of 2D TMDs [166]. The stability of these materials under ambient conditions, affecting their long-term reliability, is another area of concern [166]. 2D TMDs present exciting prospects for memory devices, significant obstacles, primarily associated with synthesis, integration, and reliability, need to be surmounted to unlock their full potential. Continued research and innovation in these areas are essential to propel the application of 2D TMDs in memory devices forward.

#### Enhancement of Memory Speed, Data Retention, Endurance, and Extinction Ratio of Memory Devices

Examination of the role and significance of memory operation speed, data retention, endurance, and extinction ratio in transistor performance, particularly in memory-intensive applications. The performance of transistors in memory-intensive applications is fundamentally tied to several key parameters, including memory operation speed, data retention, endurance, and extinction ratio. The distinctive properties of 2D TMDs present new opportunities for optimizing these parameters, thereby improving memory performance.

Memory operation speed plays a critical role in determining the performance of memory devices. The higher the operation speed, the more rapidly the device can read and write data, which is indispensable for high-performance computing applications. Recent research has indicated that floating-gate field-effect transistors (FGFETs) based on 2D TMDs, such as InSe and MoS_2_, can facilitate ultra-high-speed memory device operation, approaching the theoretical nanosecond limit [38, 308, 310, 311]. However, more research is necessary to fully optimize operation speed while reducing fabrication complexity [309]. Data retention, the capacity of a memory device to maintain stored data over time, is another vital parameter. This attribute is particularly crucial for non-volatile memory devices, expected to retain data even when power is removed. Studies suggest that FGFETs based on InSe and MoS_2_ [38, 308], polarized tunneling transistors (PTTs) based on MoS_2_ [309], and charge trap memory (CTM) devices based on WSe_2_ [313] can significantly enhance data retention characteristics, making them suitable for applications requiring long-term data storage. However, environmental factors like temperature and humidity can influence this property, demanding a comprehensive understanding of its dependence on operating conditions. Endurance, the capacity of a memory device to endure repeated read/write operations without degradation, is also an essential performance indicator. Enhanced endurance can prolong the memory devices’ lifespan, mitigating the need for frequent replacements. Research indicates that 2D TMD-based transistors [38, 308, 309, 313] can augment the cycle endurance of memory devices, chiefly due to the stability of the TMD layers under repeated charge/discharge cycles. Nevertheless, device reliability and endurance remain active areas of research. Finally, the extinction ratio, measuring the contrast between the ‘on’ and ‘off’ states of a memory device, is a crucial parameter for multi-bit storage applications. 2D TMDs, with their high on/off ratios, offer excellent extinction ratios. A high extinction ratio enables the storage of more than one bit of information per cell, facilitating ultra-high-density information storage. Recent studies have demonstrated that 2D TMD-based FGFETs can achieve an extinction ratio up to 10^10^, paving the way for multi-bit storage [308]. However, achieving consistent extinction ratios across devices is a challenge that requires attention.

The unique properties of 2D TMDs present significant opportunities to enhance the key parameters determining transistor performance in memory-intensive applications. Nonetheless, further research is required to fully harness the potential of these materials and address existing challenges.

#### Strategies in Improving Memory Speed, Data Retention, Endurance, and Extinction Ratio of Memory Devices

**Fig. 17:**
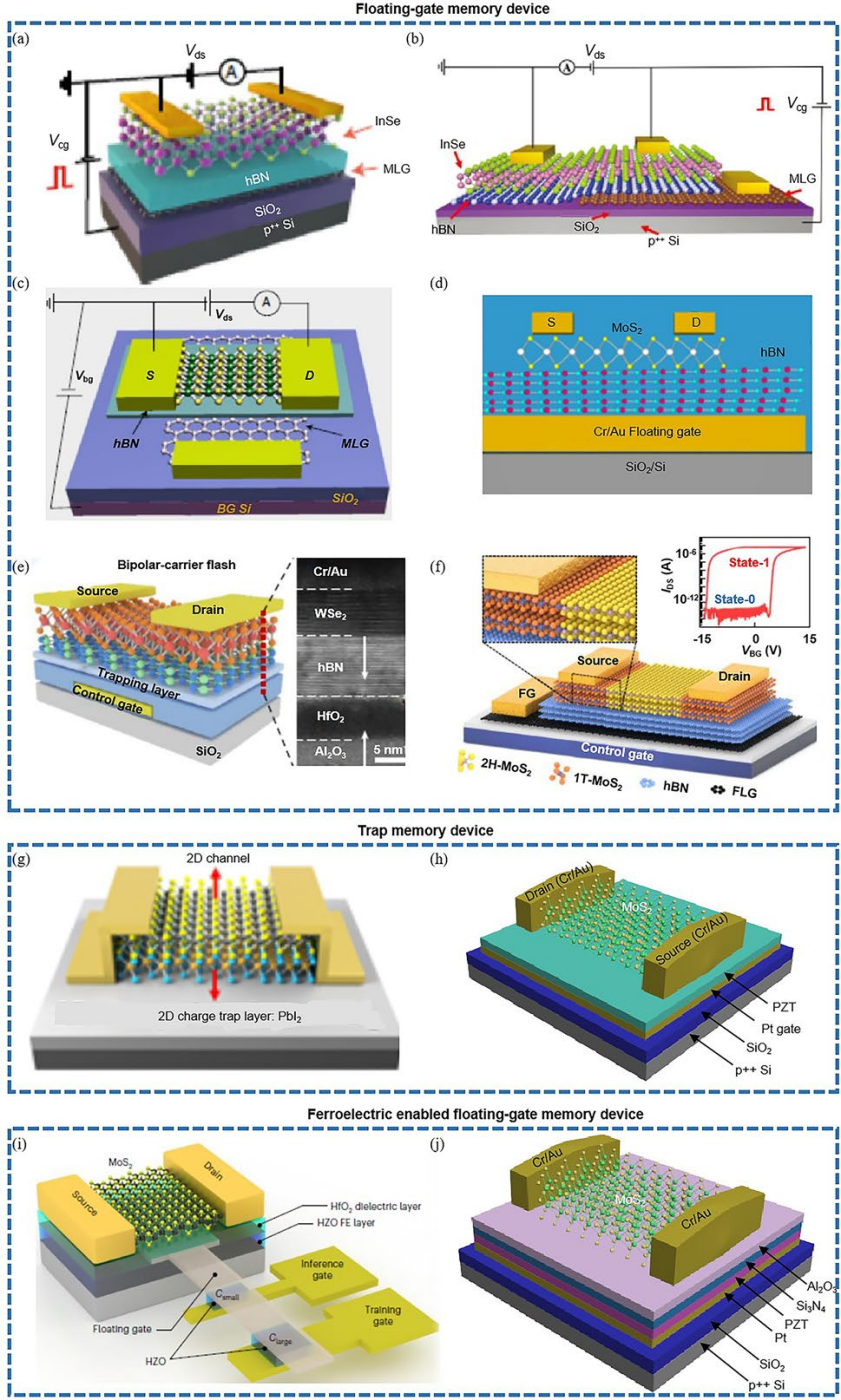
Ultrafast memory devices. **a** The floating-gate memory device with atomically sharp interface. Reproduced with permission. Reference [308] Copyright 2021, Springer Nature. **b** Ultrafast semi-floating gate homojunctions. Reproduced with permission. Reference [314] Copyright 2023, Wiley-Blackwell. **c** Ultrafast flash memory based on van der Waals heterostructures. Reproduced with permission. Reference 38] Copyright 2021, Springer Nature. **d** Ultrafast MoS_2_ floating memory. Reproduced with permission. Reference [315] Copyright 2022. **e** Ultrafast WSe_2_ bipolar flash memory. Reproduced with permission. Reference [316] Copyright 2023, Springer Nature. **f** Ultrafast flash memory based on phase-engineered edge contacts. Reproduced with permission. Reference [82] Copyright 2015, Springer Nature. **g** Ultrafast van der Waals memory with PbI_2_ as the charge trap layer. Reproduced with permission. Reference [313] Copyright 2023, Wiley-Blackwell. **h** Polarized Tunneling Transistor for Ultrafast Memory. Reproduced with permission. Reference [309] Copyright 2023, American Chemical Society. **i** Ultrafast flash memory based on a duplex two-dimensional material structure. Reproduced with permission. Reference [317] Copyright 2023, Springer Nature. **j** PZT-enabled MoS_2_ floating gate transistor for ultrafast flash memory. Reproduced with permission. Reference [310] Copyright 2023, American Chemical Society

Several strategies are currently under investigation to enhance key parameters—namely memory operation speed, data retention, endurance, and extinction ratio—of transistors based on 2D TMDs (Fig. 17), including floating-gate memory device, trap memory device, and ferroelectric enabled floating-gate memory device.

To boost memory operation speed, researchers are employing diverse strategies. Floating-gate memory device: For instance, Wu et al. exploited atomically sharp interfaces in InSe/BN/Graphene van der Waals heterostructures to achieve an ultra-high-speed memory device operation of approximately 20 ns, nearing the theoretical limit [308, 314] (Fig. 17a, b). Additionally, Liu et al. fabricated ultrafast non-volatile flash memory based on MoS_2_/BN/Graphene van der Waals heterostructures, obtaining an erase/program speed of about 20 ns due to a clean interface, a satisfactory gate coupling ratio, and an appropriate barrier height [38] (Fig. 17c). The same group has replaced the graphene floating gate using the Cr/Au floating gate in van der Waals heterostructure floating gate transistor, which can also achieve the ultra-high-speed of ~ 20 ns [315] (Fig. 17d). The same group has also made the bipolar FGFET based on WSe_2_ channel layer, achieving ultrafast n/p program speed of 20–30 ns [316] (Fig. 17e). Trap memory device: In another study, Jing Chen et al. has proposed a novel transistor configuration-polarized tunneling transistor (PTT), which has no tunnel layer compared to the traditional floating FET, enabling ultrafast operation speed of ~ 20 ns [309]. Ferroelectric enabled floating-gate memory device: A significant achievement in ultrafast memory studies is the realization of duplex device structure based on a ferroelectric field-effect transistor, which has reached the operation speed of 4.8 ns [317] (Fig. 17i). However, challenges such as complex fabrication processes and the intricacy of preparing certain materials like Pb[Zr_0.2_Ti_0.8_]O_3_ (PZT) persist in these ultrafast memories.

To enhance data retention, many strategies have focused on engineering the clean interface between the 2D TMDs [38, 308, 318]. This minimizes charge trap sites, thus improving data retention. Trap memory device: Additionally, the polarity effect of PZT in polarized tunneling transistor (PTT) memory [309], and the abundant charge traps of PbI_2_ in charge trap memory (CTM) devices can both improve data retention time [313]. However, maintaining excellent interface quality during device fabrication and obtaining high-quality PZT and PbI_2_ remain significant challenges. To augment endurance, recent research has concentrated on the development of non-volatile memory devices based on 2D TMDs that demonstrate superior charge-trapping capabilities. Various configurations, such as FGFETs [32, 38, 82, 308, 314, 315, 319, 320] (Fig. 17f), PTT [309] (Fig. 17h), and CTM [313] (Fig. 17g), with endurance exceeding 10^4^ cycles, have been demonstrated. Yet, the challenge lies in the high programming voltages often required, which may limit the practical utility of such devices. To enhance the extinction ratio, research has focused on designing atomically sharp interfaces in FGFET and optimizing dielectric-layer thickness, offering better control over the channel's on/off state. Floating-gate memory device: The improved FGFET has achieved an extinction ratio of 10^10^, currently the highest record [308]. While this extraordinary performance has opened up new multi-bit storage capabilities, these refined designs often increase device complexity and pose fabrication challenges due to their need for high yield and consistency.

In general, we think trap memory devices are more compatible and feasible for large-scale production.

Current strategies demonstrate promise for improving the performance parameters of 2D TMD-based memories, significant challenges remain. Sustained research efforts are needed to overcome these obstacles and pave the way for memory-intensive applications utilizing 2D TMDs [321].

#### Reduction of Energy Consumption in Memory Devices as Artificial Synapses

The era of artificial intelligence (AI), driven by artificial neural networks, is approaching rapidly. The massive computational demands for training these networks result in significant power consumption. As the field of AI continues to evolve, power consumption is predicted to account for one-fifth of the world's total usage [322]. Therefore, reducing AI's power consumption is of critical practical importance. The key to reducing AI's power usage lies in minimizing the power consumption of artificial neural networks. Artificial synapses, the essential components of these networks, contribute substantially to overall power consumption. As such, the reduction of power consumption in artificial synapses has become a focal point of research.

Artificial synapses mimic the functionality of biological synapses [323], transmitting signals between neurons. In this context, energy efficiency is of paramount importance. It is not merely about replicating the behavior of biological synapses, but doing so with minimal energy usage. Biological synapses operate with remarkable energy efficiency, and emulating this in artificial systems—consisting of millions or even billions of synapses—has significant implications for overall power consumption [324–326]. Memory devices, typically employed as artificial synapses, highlight the importance of 2D TMDs-based memory devices. These are emerging as promising materials for constructing low-energy, high-density artificial synaptic devices, thanks to their unique properties [327, 328]. First, the highly tunable bandgap of 2D TMDs allows for efficient control of charge flow, a crucial feature in reducing energy consumption in memory devices [98]. Second, the ultrathin nature of 2D TMDs facilitates lower leakage currents, contributing to improved energy efficiency [60]. Third, the atomic-scale thickness of 2D TMDs facilitates rapid heat dissipation and swift responses to external stimuli, both of which are crucial for energy-efficient operation [329]. Moreover, 2D heterostructures provide a wealth of device operations and physics [330].

Recent advancements have demonstrated low energy-consumption artificial-synapse configurations using 2D TMDs, such as FGFET [318, 331], heterostructure FET [332], and ferroelectric FET [333]. However, challenges persist, including the reliable control of doping levels and ensuring stable operation under varying conditions [269]. Synthesizing 2D TMDs for large-scale applications remains a significant issue. Techniques such as chemical vapor deposition (CVD) and metal–organic chemical vapor deposition (MOCVD) have been used to produce high-quality 2D materials, but these methods have limitations, including natural defects during the synthesis process and inefficient growth speed [334, 335]. The importance of energy efficiency in memory devices serving as artificial synapses is paramount. As we continue to advance in the field of neuromorphic engineering, 2D TMDs-based transistors emerge as a promising path towards realizing highly efficient artificial synapses [336]. However, further research is necessary to overcome remaining challenges and fully exploit the potential of these materials.

#### Strategies in Energy Consumption Reduction

In addressing the urgent need for highly energy-efficient artificial synapses, various strategies employing 2D-based transistors are being explored (Table 2) [310, 318, 319, 332, 333, 337–341]. This article discusses their effectiveness and associated challenges. Currently, two primary methods are used to calculate the power consumption of single weight-updating behavior in artificial synapses.

**Table 2:** Comparison of representative synaptic devices based on 2D transistors

Channel layer	Weight control layer	Nonlinearity *NL*_*LTP*_*/NL*_*LTD*_	Retention time (s)	Write pulse	Switch energy	Recognition efficiency	References
SnS_2_/11 nm	h-BN/ FLG	0.7/0.8	1 × 10^4^	− 6 V/50 ms	7 pJ	90%	[309]
MoS_2_/2.8 nm	h-BN/ FLG	~	5 × 10^2^	40 V/10 ms	2.52 fJ	92%	[318]
MoS_2_/5.8 nm	VP	2/− 2	~	5 V/40 ns	> 2.2 pJ	95.2%	[332]
InSe/13.8 nm	InO_x_	~	1 × 10^4^	− 40 V/50 ms	~	70%	[337]
MoS_2_/2.1 nm	h-BN/ graphene	1.7/ ~	3.5 × 10^2^	3 V/0.1 s	5 fJ	~	[338]
WSe_2_/10 nm	HfO_x_/HfO_2_	0.35/0.05	2 × 10^3^	− 40 V/100 ms	~	88%	[339]
MoTe_2_/7 nm	Sr-SiN_x_	0.81/0.72	1 × 10^4^	− 40 V/4 s	8 nJ	91%	[340]
BP/12 nm	P(VDF-TrFE)	4.47/2.07	4 × 10^3^	− 25 V/100 ms	41 fJ	93.6%	[333]
MoS_2_/2 nm	W	0.7/− 0.8	1 × 10^4^	− 7.5 V/0.5 ms	~	94%	[341]
MoS_2_/2.8 nm	Si_3_N_4_	0.025/0.042	1 × 10^4^	− 8 V/40 ns	0.0003 fJ	97.3%	[329]

The first method is to calculate the lowest dynamic energy consumption of a single weight-updating behavior in an artificial synapse device using the formula: E = *I*_ds-peak_ × *V*_ds_ × *t*_pulse_ [303, 310, 318, 331, 333]. Here, *I*_ds-peak_ represents the peak source–drain current under voltage-pulse stimuli, *V*_ds_ is the source-drain voltage, and *t*_pulse_ is the width of voltage-pulse stimuli. A few studies have adopted this method to derive the lowest energy consumption. In a study by Yanan Wang et al., an FGFET based on SnS_2_/BN/Graphene was fabricated and used as artificial synapses for neuromorphic computing. The researchers obtained the lowest dynamic energy consumption (7 pJ) by selecting the smallest values for *I*_ds_, *V*_ds_, and *t*_pulse_. However, the fabrication process involved multiple precise alignments and transfers, increasing the complexity of fabrication [318]. In another study, Zhaoying Dang et al. prepared black phosphorus/Ferroelectric P(VDF-TrFE) Field-Effect Transistors for use as artificial synapses in constructing neural networks for neuromorphic computing. Despite achieving a low final dynamic energy consumption (41 fJ), the work involved the use of organic ferroelectric material (P(VDF-TrFE)), which struggles to maintain stable ferroelectric properties [333]. Ruixuan Peng et al. investigated a single gate-voltage-programmed MoTe_2_ transistor, in which the transistor’s polarity-switchable feature is controlled by the difference between drain and gate voltages. The reconfigurable transistor was used as an artificial synapse, achieving a single weight-updating dynamic energy consumption of 0.73 fJ [303]. Jing Chen et al. employed PZT-Enabled MoS_2_ Floating Gate Transistors as artificial synapses for low energy-consumption neuromorphic computing. Despite reaching a record low energy consumption (0.0003 fJ), the work involved the use of ferroelectric PZT material, complicating the fabrication process [310].

The second method is to calculate the lowest dynamic energy consumption of a single weight-updating behavior in an artificial synapse device using the formula: E = (*V*^2^ × Δ*G* × *t*_pulse_)/N_pulse_ [332, 341], where *V* represents the training voltage, Δ*G* is the conductance change during training, and *t*_pulse_ and N_pulse_ are the width and number of pulses used for training, respectively. Some studies have chosen this method to record the lowest energy consumption. Xiaoxian Liu et al. constructed a violet phosphorus (VP)/MoS_2_ heterostructure transistor, where VP is used for trapping carriers and MoS_2_ is the channel layer, achieving a low energy consumption of 2.2 pJ. However, the use of VP, which is unstable in atmospheric conditions, presents challenges [332]. Heng Xiang et al. prepared a MoS_2_-based FGFET, where MoS_2_ is the channel layer, Al_2_O_3_ is the tunnel layer, W is the floating layer, and Hf_0.5_Zr_0.5_O_2_ is the blocking layer. Despite achieving a low average energy consumption in a single synaptic event, fabricating the high-quality ferroelectric layer of Hf_0.5_Zr_0.5_O_2_ proved difficult [341].

These strategies present promising avenues for reducing energy consumption in 2D TMD-based memory devices serving as artificial synapses, they also pose significant challenges. Overcoming these challenges will necessitate further research and innovation in material synthesis, device fabrication, and device operation.

## Conclusions and Prospects

### Conclusions

#### Concise Recapitulation of the Key Points Discussed in the Review, Emphasizing the Importance of Dimension and Performance Boundaries

This review offers a comprehensive examination of the dimensional and performance constraints of transistors developed using 2D TMDs. The unique layered structure of 2D TMDs has paved the way for atomic-scale dimensional manipulation [3]. This review discusses efforts to miniaturize channel length, reduce gate length, decrease source/drain contact length, and shrink dielectric thickness, each of which substantially influences the overall transistor dimensions [1, 87].

We also delve into the performance limits of transistors based on 2D TMDs, with particular attention paid to the tunable bandgap of TMDs. This characteristic significantly affects transistor performance and scalability. We overview strategies for diminishing source/drain contact resistance and subthreshold swing, both central to performance and power consumption. The significance of reducing hysteresis, augmenting carrier mobility, enhancing the on/off ratio, and the potential for realizing p-type transistors and single logic transistors are also emphasized, as each factor impacts device reliability, speed, and energy efficiency.

Additionally, the review investigates the performance boundaries of 2D TMD-based transistors as memory devices, discussing the merits and challenges of utilizing 2D TMDs in this context. The influence of memory operation speed, data retention, endurance, and extinction ratio on performance is examined, alongside an overview of strategies for improving these parameters. The importance of energy efficiency and strategies for reducing energy consumption in memory devices as artificial synapses were also highlighted.

Throughout the review, the importance of dimension and performance boundaries of 2D TMDs-based transistors was emphasized. The exploration of these boundaries is not only significant for understanding the fundamental properties of 2D TMD-based transistors but also crucial for future technological advancements, although challenges remain in realizing the full potential of these devices.

#### Discussion on How the Findings and Discussions in the Review Might Impact Future Research Directions or Applications

The insights gleaned from this review on the dimension and performance limits of transistors based on 2D TMDs could significantly influence future research directions and applications. The exploration of the dimension and performance limits of these transistors provides a roadmap for researchers to further optimize the design and fabrication processes.

Firstly, the capability to manipulate dimensions at the atomic scale presents a promising avenue for further miniaturization of electronic devices [1]. The exploration of strategies for the reduction of channel length, gate length, source/drain contact length, and dielectric thickness could stimulate research focused on developing innovative approaches to achieve further scaling down. The performance limits of 2D TMD-based transistors, as discussed in this review, underscore areas necessitating further exploration. The tunable bandgap of 2D TMDs and its influence on transistor performance and scalability, herald new research opportunities. This could yield devices with superior performance parameters, encompassing reduced contact resistance, subthreshold swing, hysteresis, and enhanced carrier mobility and on/off ratio. Future research could concentrate on devising more effective strategies in these areas.

Another pivotal aspect is the potential use of 2D TMD-based transistors in memory devices. The insights from this review on enhancing memory operation speed, data retention, endurance, and extinction ratio, along with strategies for energy consumption reduction, could guide research towards creating efficient, high-performance memory systems. Moreover, the realization of p-type transistors and single logic transistors using 2D TMDs, as discussed in this review, constitutes a significant breakthrough with extensive applications. These findings could stimulate further research on the development of CMOS technology and efficient logic operations using 2D TMDs.

Furthermore, the examination of the interplay between dimension and performance limits could facilitate the development of theoretical models and simulation tools to predict the performance of 2D TMD-based devices [28, 213]. Such tools could considerably accelerate the design and optimization process, thus clearing the path for rapid advancements in the field. Finally, the findings from this review could stimulate a multitude of research directions and applications in the 2D TMD domain. The ongoing exploration of these materials could potentially revolutionize the fields of electronics, optoelectronics, and energy storage, if the dimension and performance limits are successfully overcome [166].

### Prospects

#### Discuss the Potential Impact on Industries and Technologies (e.g., Electronics, Optoelectronics, Energy Storage) If the Dimension and Performance Limits of 2D TMD-Based Transistors are Successfully Overcome

Surmounting the dimensional and performance limits of transistors based on 2D TMDs could create significant impacts across a variety of industries, including electronics, optoelectronics, and energy storage [1, 87].

In the electronics industry, successful optimization of 2D TMD-based transistors could pave the way for a new generation of miniaturized, high-performance devices operating at low voltages [1]. Such advancement could revolutionize the design and manufacturing processes of electronic devices, ushering in a new age of energy-efficient and powerful gadgets. The potential for stable operation of these transistors could further illuminate the prospects for 2D TMD electronics, creating new opportunities for the development of sophisticated electronic systems [6, 87]. In the realm of optoelectronics, which merges photonics with electronics, overcoming the limitations of 2D TMD-based transistors could stimulate the development of more efficient and compact optoelectronic devices [349, 350]. This could revolutionize various applications, encompassing communication systems, data storage devices, and sensors, and also contribute to the development of advanced photodetectors, photovoltaics, and light-emitting diodes (LEDs) [350]. Within the energy storage industry, enhanced performance of 2D TMD-based transistors could facilitate the fabrication of efficient and compact energy storage devices. This could significantly influence the advancement of renewable energy technologies, leading to more sustainable and efficient energy systems [351]. Additionally, TMD-based supercapacitors could provide high power delivery and extended cycle life, making them ideal for applications necessitating rapid energy discharge.

Moreover, successful enhancement of 2D TMD-based transistors could bolster the sustainability performance of various industries. For instance, in the plastic and petrochemical sectors, the adoption of advanced technologies such as 2D TMD-based transistors could lead to process optimization and improved environmental sustainability [342]. Furthermore, overcoming the dimensional and performance limits of 2D TMD-based transistors could benefit emerging technologies like wearable electronics and Internet of Things (IoT) devices. The mechanical flexibility and superior electrical properties of these materials could facilitate the integration of these transistors into advanced, flexible, lightweight, and energy-efficient devices [10].

Overcoming the dimensional and performance boundaries of 2D TMD-based transistors could profoundly influence various industries and technologies. This could drive the development of more efficient, powerful, and sustainable systems, thereby fueling the next industrial revolution.

#### Highlighting Anticipated Challenges, Opportunities, and Potential Solutions in the Exploration of Dimension and Performance Boundaries of 2D Transistors

One primary challenge resides in the fabrication process, where generating high-quality, defect-free 2D materials and integrating them into devices proves to be a complex task [60]. This difficulty is especially pronounced in large-scale production, a crucial factor for commercial applications [330]. Nevertheless, these challenges also foster opportunities for developing innovative fabrication and synthesis methods. For instance, recent advancements in CVD show promise in the scalable production of 2D materials [347, 352].

Moreover, the controllable polarity growth of 2D TMDs poses a significant challenge. Most transistors based on 2D TMDs are n-type, which precludes the fabrication of CMOS circuits. Thus, achieving controllable polarity growth of 2D TMDs is of paramount importance [353]. Environmental stability of 2D materials poses another challenge as many of them are susceptible to air, moisture, and temperature fluctuations. These adversities could be alleviated through encapsulation techniques or by developing more robust materials [18]. The performance of 2D transistors also raises concerns. Parameters such as contact resistance, subthreshold swing, and device stability require enhancement [1, 87]. To address these issues, research could concentrate on optimizing device architecture [35] and exploring alternative contact materials [31]. The dielectric layer's impact on device performance presents another hurdle. The choice of this component can significantly influence transistor performance, necessitating meticulous selection and optimization [121]. Pioneering research has already evidenced the feasibility of transistors with high-κ material, thereby opening new avenues to explore advanced electronic devices [30, 217]. Theoretical and computational modeling offers an opportunity to push the performance boundaries of 2D transistors. The development of precise models and simulations can guide experimental strategies and accelerate design and optimization processes [23, 28, 174].

The exploration of dimension and performance boundaries of 2D transistors involves significant challenges, it also presents numerous opportunities and potential solutions. Notably, leading semiconductor companies such as Taiwan Semiconductor Manufacturing Company (TSMC) and Intel have been focusing on 2D TMDs in recent years [343–346, 346, 348, 355]. Continued investigation and development of 2D materials could lead to groundbreaking advancements across various technologies.
